# Targeted elimination of senescent cells by engineered extracellular vesicles attenuates atherosclerosis in ApoE^-/-^ mice with minimal side effects

**DOI:** 10.7150/thno.87484

**Published:** 2023-09-18

**Authors:** Liang Zhang, Chen Wang, Wei Hu, Te Bu, Wenqi Sun, Tian Zhou, Shuo Qiu, Mengying Wei, Helin Xing, Zhelong Li, Guodong Yang, Lijun Yuan

**Affiliations:** 1 Department of Ultrasound Diagnostics, Tangdu Hospital, Fourth Military Medical University, Xi'an, People's Republic of China.; 2 State Key Laboratory of Holistic Integrative Management of Gastrointestinal Cancers, Department of Biochemistry and Molecular Biology, Fourth Military Medical University, Xi'an, People's Republic of China.; 3 Department of Prosthodontics, Beijing Stomatological Hospital and School of Stomatology, Capital Medical University, Beijing, 100050, China.

**Keywords:** atherosclerosis, extracellular vesicles, cellular senescence, magnetic nanoparticles, BCL-2-associated X protein

## Abstract

Senescent cells in plaques emerge as a detrimental factor for atherosclerosis (AS), for which targeted senolysis might be a promising therapeutic strategy. The development of safe and efficient senolytics for senescent cell eradication by targeted delivery is greatly needed.

**Methods:** Pro-apoptotic intelligent *Bax* (i*Bax*)-overexpressing plasmid was constructed by molecular cloning, in which *Bax* CDS was fused to miR-122 recognition sites. Extracellular vesicle-based senolytics (EV^iTx^) were developed to be conjugated with magnetic nanoparticles on the surface, i*Bax* mRNA encapsulated inside, and BAX activator BTSA1 incorporated into the membrane. EV^iTx^ was characterized, and *in vivo* distribution was tracked *via* fluorescence imaging. The therapeutic effects of EV^iTx^ on AS and its systemic side effects were analyzed in ApoE^-/-^ mice.

**Results:** Magnetic nanoparticles, i*Bax* mRNA and BAX activator BTSA1 were efficiently loaded into/onto EV^iTx^. With external magnetic field navigation, EV^iTx^ was delivered into atherosclerotic plaques and induced significant apoptosis in senescent cells regardless of origins. Repeated delivery of EV^iTx^ *via* tail vein injection has achieved high therapeutic efficacy in ApoE^-/-^ mice. Notably, EV^iTx^ is inevitably accumulated in liver cells, while the i*Bax* mRNA was translationally repressed by miR-122, an endogenous miRNA highly expressed in hepatocytes, and thus the liver cells are protected from the potential toxicity of *Bax* mRNA.

**Conclusion:** Our work demonstrated that magnetic EV-based delivery of i*Bax* mRNA and the BAX activator BTSA1, efficiently induced apoptosis in recipient senescent cells in atherosclerotic plaques. This strategy represents a promising treatment approach for AS and other age-related diseases.

## Introduction

Atherosclerosis (AS) is a prevalent vascular disease characterized by dyslipidemia and chronic inflammation 1, 2. Despite the use of preventive lipid-lowering and anti-inflammatory therapeutic strategies, there is still a critical need for more effective treatment options 3, 4. Recent research has revealed a significant accumulation of senescent cells in plaques positively correlated with plaque instability 5, 6. Senescent cells in plaques aggravate chronic inflammation and accelerate AS progression generating a senescence-associated secretory phenotype (SASP) consisting of matrix remodeling proteases, chemokines, cytokines, growth factors, and lipids 7, 8. Therefore, the targeted removal of senescent cells in plaques presents a promising therapeutic strategy for treating AS 9, 10.

Senescent cells acquire resistance to apoptosis by upregulating pro-survival pathways, such as that of B cell lymphoma-2 (BCL-2) family 11, 12. Currently, the clearance of senescent cells, termed senolysis, is primarily achieved by inhibiting pro-survival pathways (“inhibit the inhibitors”) 13-16. However, senolytic drugs, such as dasatinib (D), quercetin (Q), and navitoclax (N), have limitations in their ability to target specific types of senescent cells 17. A combination of drugs directed against single targets or a drug with multiple pro-survival targets may be necessary to improve the effectiveness of cellular clearance 13. However, the combination approach instigates significant side effects, including thrombocytopenia and neutropenia 18.

BCL-2 associated X protein (BAX), a natural inhibitor of the pro-survival protein, is a key pro-apoptotic protein and plays an essential role in regulating the mitochondrial apoptotic pathway 19, 20. Increasing the intracellular active BAX level may trigger apoptosis in broad-spectrum senescent cells regardless of origin 21. Therefore, delivering *Bax* mRNA and the BAX activator BTSA1 to senescent cells may represent a novel approach for clearing senescent cells (“activate the activator”).

Superparamagnetic iron oxide nanoparticles (SMN) have recently gained significant attention for targeted drug delivery due to their advanced targeting capacity, biodegradability, biological compatibility, and low toxicity 22. The ability for magnetic targeting is not dependent on cell types but on the recognition of the spatial location of the affected tissue, rendering it a suitable method for delivering drugs to senescent cells in plaques 23-27. Furthermore, small extracellular vesicles (EVs) with a diameter ranging from 30 to 150 nm exhibit favorable biocompatibility and cycling stability, and are well-suited for carrying protein and nucleic acid-based drugs 28. Thus, *Bax* mRNA-loaded EVs modified with SMN (EV^SMN^) hold significant potential as drug carriers for treating AS.

Even for targeted delivery, nanoparticles, including EVs, are accumulated in the liver, causing liver injury when *Bax* mRNA is excessively delivered 29, 30. Therefore, repressing *Bax* translation in liver cells is imperative to avoid potential toxicity. MicroRNAs are key regulators of gene expression that destabilize target mRNAs or inhibit their translation 31. miR-122-5p (miR-122), is a liver-specific molecule with an estimated cellular abundance of 50,000-82,000 copies in adult liver cells 32, 33, suggesting that *Bax* mRNA harboring miR-122 recognition sites in the 3'-untranslated region (3'-UTR) (termed as *iBax*) could be translationally repressed in liver cells.

Herein, a therapeutic EV (EV^iTx^) was engineered with SMN conjugated on the surface, i*Bax* mRNA encapsulated inside and BAX activator BTSA1 incorporated into the membrane. With external magnetic field (MF) navigation, EV^iTx^, when targeted to atherosclerotic plaques, induced significant apoptosis in senescent cells regardless of origin. Notably, when delivered into liver cells, i*Bax* mRNA was translationally repressed by miR-122 endogenously expressed in liver cells and thus had minimal hepatotoxicity. Repeated delivery of EV^iTx^ *via* tail vein injection achieved high therapeutic efficacy and low side effects in ApoE^-/-^ mice. Hence, the EV^iTx^-based strategy offers a promising treatment approach for AS and other age-related diseases.

## Results

### Construction and characterization of SMN-decorated EVs (EV^SMN^)

**Figure 1:**
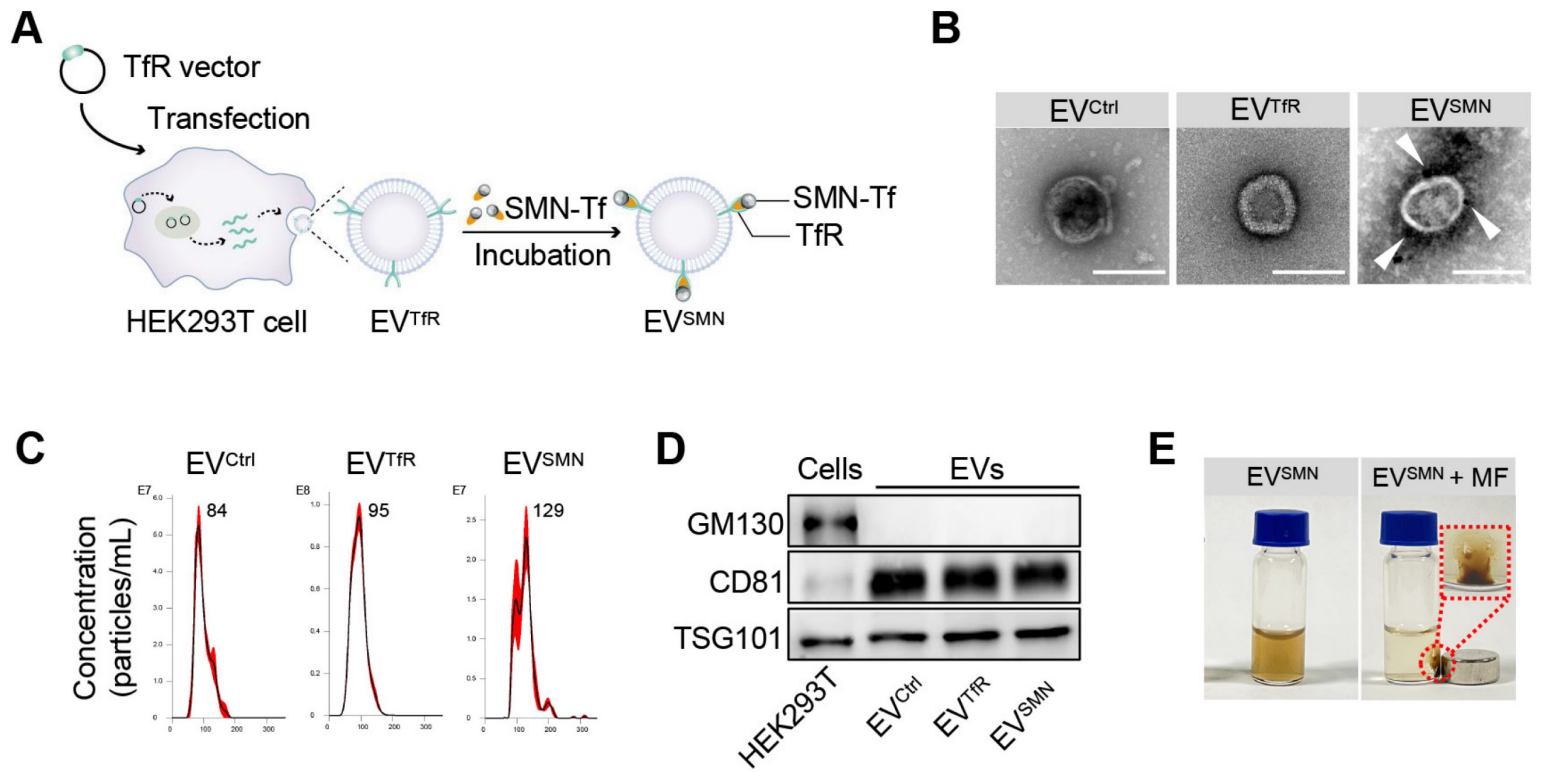
** Construction and characterization of EV^SMN^.** (A) Schematic illustration of EV^SMN^ synthesis. HEK293T cells were transfected with the TfR vector, which secretes EVs with high expression of TfR on the membrane surface (EV^TfR^). SMN-Tf was co-incubated with EV^TfR^, resulting in the attachment of SMN-Tf to the surface of EV^TfR^ through the interaction between TfR and Tf. (B) Representative TEM images of the indicated EVs. White arrows represent SMN-Tf. Scale bar = 100 nm. (C) Size distribution of EV^Ctrl^, EV^TfR^, or EV^SMN^. (D) Western blot of the exclusive and inclusive EV markers in isolated EVs and parental cells. (E) Digital images of EV^SMN^ before and after exposure to MF *in vitro*. The data shown are representative images of three different experiments.

EVs were first decorated with SMN-Tf (EV^SMN^) to endow them with magnetic targeting ability. Specifically, transferrin receptor (TfR)-expressing EVs (EV^TfR^) were co-incubated with transferrin (Tf)-modified SMN (SMN-Tf) to obtain magnetized EV^SMN^ through the TfR-Tf interaction (Figure 1A). A TfR overexpression vector was designed and transfected into HEK293T cells, resulting in engineered EV^TfR^ with high expression of TfR on the membrane surface (Figure S1). Commercially available SMNs were employed as the starting material to fabricate SMN-Tf. The COO^-^ groups located on the surface of SMN were activated using carbodiimide (EDAC) and N-hydroxysulfosuccinimide sodium salt (sulfo-NHS) to facilitate their interaction with the NH^3+^ of Tf, leading to the successful attachment of Tf onto the surface of SMN, producing SMN-Tf (Figure S2A). Transmission electron microscopy (TEM) showed that SMN-Tf and SMN had similar sizes and morphologies (Figures S2B and S2C).

Subsequently, SMN-Tf was incubated with EV^TfR^ at 4°C in a rotary mixer, resulting in the formation of EV^SMN^, which was isolated through magnetic separation. The morphological features of EV^SMN^ were examined using TEM, revealing dark spots surrounding spherical vesicles, providing strong evidence for the successful coupling of SMN-Tf with EV^SMN^ (Figure 1B). Nanoparticle tracking analysis (NTA) showed that EV^Ctrl^ (EVs secreted by HEK293T cells transfected with empty vector plasmids), EV^TfR^, and EV^SMN^ had similar size distributions, with diameters ranging from 30-150 nm (Figure 1C). Western blot analysis showed that the expression patterns of inclusive and exclusive EV markers in donor and derived EVs were similar between the EV^SMN^ and the control groups (Figure 1D). When the MF was applied, well-dispersed EV^SMN^ in PBS converged around the applied MF, demonstrating that EV^SMN^ had the magnetic targeting ability *in vitro* (Figure 1E). This data indicated the successful construction of magnetized EV^SMN^ with excellent magnetic targeting performance.

### Effective delivery of EV^SMN^ into atherosclerotic plaques with magnetic navigation

**Figure 2:**
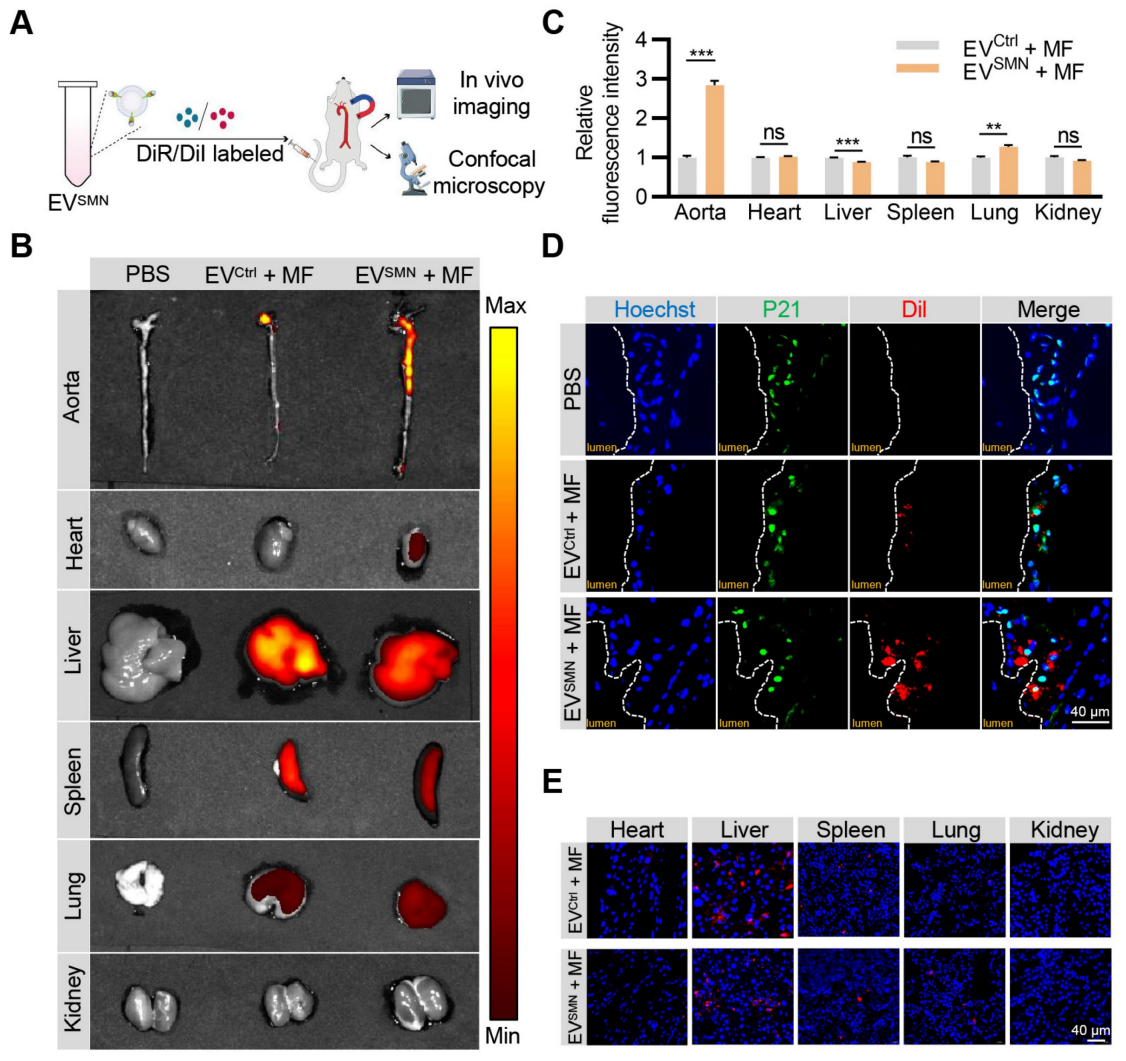
**Effective delivery of EV^SMN^ into atherosclerotic plaques with magnetic navigation.** (A) Schematic illustration of the experimental procedure. ApoE^-/-^ mice were injected with PBS or DiI/DiR-labeled EV^Ctrl^/EV^SMN^ while applying MF near the aorta. 12 h later, the aorta and vital organs (heart, liver, spleen, lung, kidney) were harvested for IVIS imaging and confocal microscopy analyses. (B) Representative IVIS images showing the distribution of DiR-labeled EV^Ctrl^/EV^SMN^ in the aorta and vital organs following intravenous administration in ApoE^-/-^ mice, with application of MF near the aorta. (C) Quantification of Figure 2B. 8-week-old male ApoE^-/-^ mice were fed a high-fat diet for 8 weeks, n = 3. (D) Representative confocal images demonstrating the localization of EV^Ctrl^/EV^SMN^ labeled with DiI in P21^+^ cells within aortic root atherosclerotic plaques. (E) Representative fluorescence microscopic images of the localization of EV^Ctrl^/EV^SMN^ labeled with DiI in vital organs. The data are representative images of three different experiments. Statistical significance determined by Student's *t*-test. ***p < 0.001. **p < 0.01. ns, no significance.

Previous studies have demonstrated that EV^SMN^ could be magnetically guided to specific lesion locations 23, 27. Therefore, we investigated whether EV^SMN^ could target atherosclerotic plaques through magnetic navigation. EV^SMN^ were labeled with DiR/DiI fluorescent dyes and systemically administered to ApoE^-/-^ mice *via* the tail vein while applying MF near the aorta. The *in vivo* imaging system (IVIS) and confocal microscopy analyses were used to visually demonstrate the enrichment of EV^SMN^ in the plaque and track the distribution of labeled EVs (Figure 2A).

IVIS imaging analysis demonstrated that unmodified EV^Ctrl^ (EV^Ctrl^ + MF group) exhibited a modest accumulation level within the aortic plaque region, consistent with previously reported findings 34, 35. Notably, the fluorescence signal intensity and area of the aortic region were markedly enhanced in the EV^SMN^ + MF group (Figures 2B and 2C). These results provided compelling evidence that magnetized EV^SMN^ possessed excellent targeting capability towards atherosclerotic plaques localized in the aorta.

Confocal microscopy analysis confirmed the co-localization of magnetized EV^SMN^ with P21^+^ cells within atherosclerotic plaques, likely consisting of senescent foamy macrophages or other senescent cell types. The extent of EV^SMN^ enrichment within these cells was significantly higher than EV^Ctrl^ (Figure 2D). These data provided a strong rationale for developing a targeted drug carrier that can effectively clear senescent cells in AS.

Despite the remarkable enhancement of EV^SMN^ enrichment in plaques by magnetic navigation, their intrinsic *in vivo* distribution properties rendered them highly enriched in other organs, particularly the liver, as evidenced by the IVIS imaging and confocal microscopy analysis (Figures 2B and 2E). Therefore, the mitigation of the hepatotoxicity associated with drug-loaded EV^SMN^ was crucial.

### Engineering of i*Bax* mRNA repressed by miR-122

**Figure 3:**
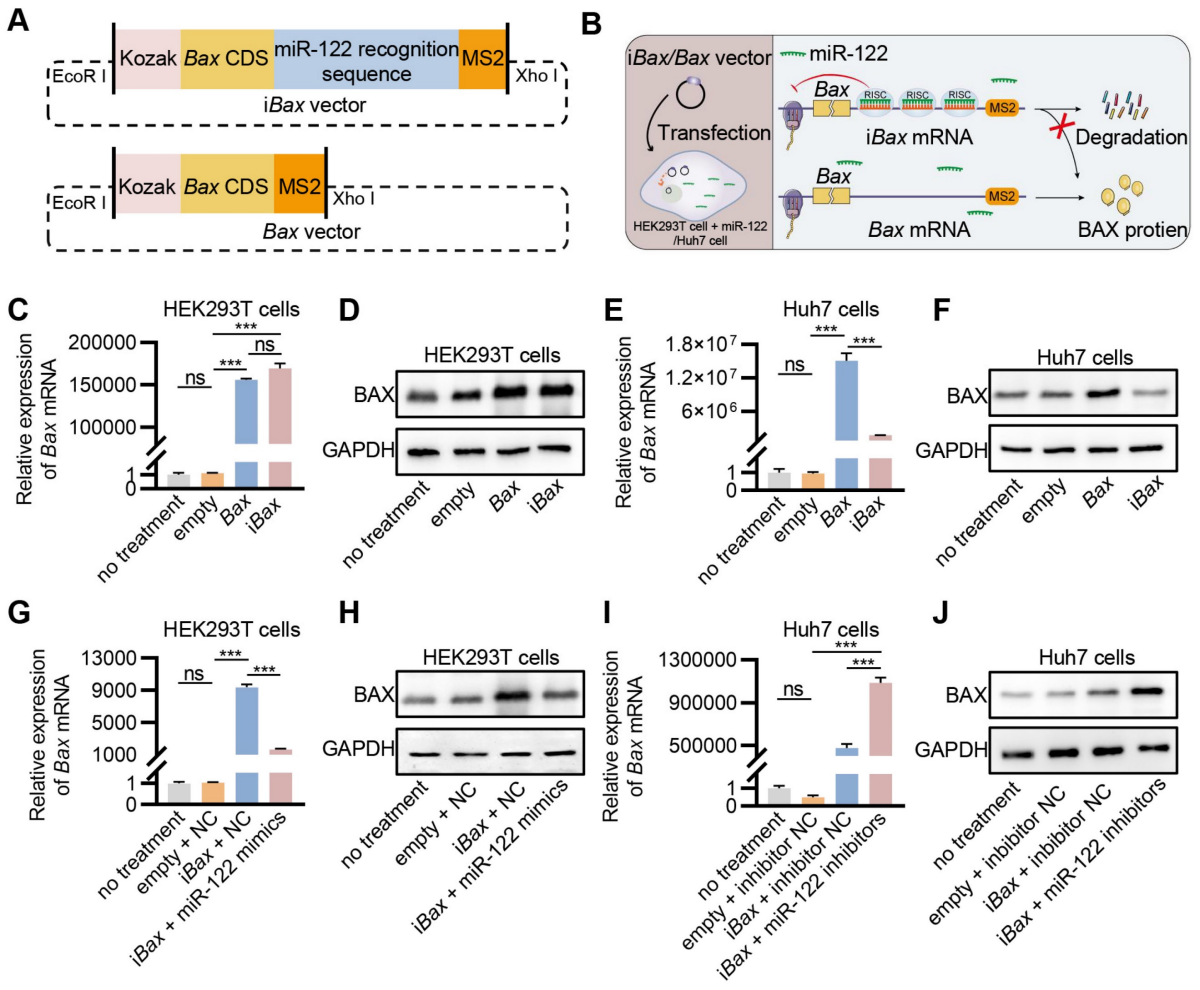
** Engineering of i*Bax* mRNA repressed by miR-122.** (A) Schematic of i*Bax* plasmid and *Bax* plasmid. (B) Schematic illustrating the specific process by which i*Bax* transcripts are synthesized and regulated by miR-122 following i*Bax* vector transfected into HEK293T/Huh7 cells. (C) qPCR analysis of *Bax* mRNA in transfected HEK293T cells. (D) Western blot analysis of BAX protein expression in HEK293T cells after transfection. (E) qPCR analysis of *Bax* mRNA in transfected Huh7 cells. (F) Western blot analysis of BAX protein expression in Huh7 cells after transfection. (G) qPCR analysis of *Bax* mRNA in HEK293T cells after transfection with *Bax*/i*Bax* vector combined with miR-122 mimics or NC. (H) Western blot analysis of BAX protein expression in HEK293T cells after transfection with *Bax*/i*Bax* vector combined with miR-122 mimics or NC. (I) qPCR analysis of *Bax* mRNA in Huh7 cells after transfection with *Bax*/i*Bax* vector combined with miR-122 inhibitors or inhibitor NC. (J) Western blot analysis of BAX protein in Huh7 cells after transfection with *Bax*/i*Bax* vector combined with miR-122 inhibitors or inhibitor NC. Data are presented as mean ± SEM of three independent experiments. Statistical significance was determined by one-way ANOVA with Tukey's post hoc test. ***p < 0.001. ns, no significance.

MicroRNAs are essential regulators of gene expression that destabilize target mRNAs or inhibit their translation 31. miR-122, a mammalian liver-specific non-coding polyadenylated RNA with an average count of 50,000-82,000 copies per cell in the adult liver 32, 33, could serve as a potential candidate for the construction of miRNA-repressed i*Bax* mRNA to circumvent BAX-related hepatotoxicity. We inserted three sequences complementary to miR-122 for cloning the i*Bax* vector. As a control, *Bax* expression vector without the miR-122 recognition site was also constructed (*Bax* vector) (Figure 3A). We hypothesized that the engineered i*Bax* mRNA would be subjected to degradation or translational repression with elevated levels of miR-122, whereas the expression of *Bax* mRNA would remain unaffected (Figure 3B). After transfection, the *Bax*/i*Bax*-treated group showed a significant increase in *Bax* mRNA and protein expression compared to the control group in HEK293T cells (Figures 3C and 3D). In contrast to HEK293T cells, the group transfected with the i*Bax* vector in Huh7 cells synthesized significantly less *Bax* mRNA and protein than that transfected with the *Bax* vector (Figures 3E and 3F). The results indicated a significant decrease in transcription and translation efficiency of the i*Bax* vector in Huh7 cells compared to the control *Bax* vector, which was not observed in HEK293T cells. This difference might be attributed to the higher levels of miR-122 in Huh7 cells than in HEK293T cells (Figure S3A).

We tested this hypothesis by transfecting miR-122 mimics into HEK293T cells; the results showed significantly upregulated intracellular miR-122 levels (Figure S3B). Co-transfection of i*Bax* with miR-122 mimics in HEK293T cells showed a considerably lower expression of *Bax* at mRNA and protein levels compared to co-transfection with i*Bax* vector with negative controls (NC) (Figures 3G and 3H). The expression level of an intracellular miR-122 inhibitor was increased in Huh7 cells to antagonize miR-122 (Figure S3C). Co-transfection of i*Bax* with miR-122 inhibitors in Huh7 cells showed a substantially higher expression of *Bax* at mRNA and protein levels than the control group (Figures 3I and 3J). These results indicated that i*Bax* mRNA was translationally blocked by miR-122.

### Encapsulation of i*Bax* mRNA into EVs

**Figure 4:**
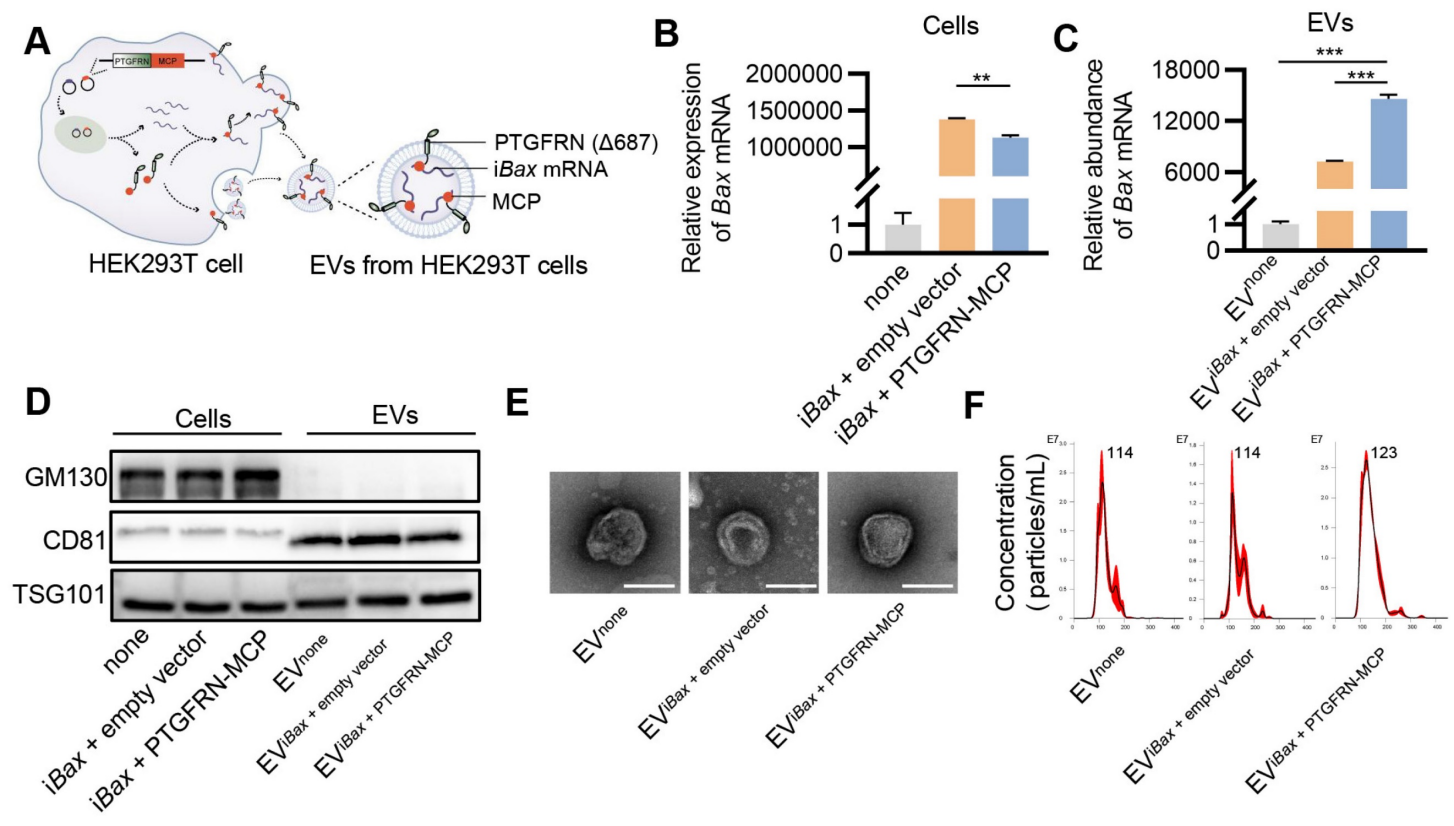
** Encapsulation of i*Bax* mRNA into EVs.** (A) Schematic of i*Bax* mRNA encapsulation by PTGFRN-MCP fusion protein-functionalized EVs. (B) Expression of *Bax* mRNA in HEK293T cells. (C) Abundance of *Bax* mRNA in EVs derived from HEK293T cells with indicated treatments. EV^none^, EVs secreted by HEK293T cells without any treatment. (D) Western blot analysis to examine the exclusive and inclusive EV markers in both isolated EVs and parental cells. The cells were transfected with either none, i*Bax* vector combined with empty vector, or i*Bax* vector combined with PTGFRN-MCP vector. (E) Representative TEM images of the indicated EVs, Scale bar = 100 nm. (F) Size distribution of EV^none^, EV^i*Bax* + empty vector^, or EV^i*Bax* + PTGFRN-MCP^. Data are presented as mean ± SEM of three independent experiments. Statistical significance was determined by one-way ANOVA with Tukey's post hoc test. **p < 0.01. ***p < 0.001.

Several studies have employed the MS2/MS2 coating protein (MCP) system to preferentially load target RNA into EVs to enhance the delivery efficacy of EV-mediated mRNA 36, 37. MS2 was modified to fuse with the target mRNA, while the MCP was fused to one of the EV scaffold proteins, such as Δ687 PTGFRN, CD9, or CD63. In this study, i*Bax* mRNA was flanked with MS2 downstream of the coding sequence, and MCP was fused to the C-terminus of Δ687 PTGFRN (PTGFRN-MCP) (Figure S4). The interaction of MS2 and MCP facilitated the sorting of mRNA into engineered EVs (Figure 4A).

Initially, we investigated whether the PTGFRN-MCP fusion protein could enhance the encapsulation of i*Bax* mRNA in EVs produced by the packaging cells. To this end, HEK293T cells were co-transfected with i*Bax* and PTGFRN-MCP vectors, or the empty vector, and i*Bax* mRNA was analyzed by qPCR. The PTGFRN-MCP fusion protein exhibited a significant ability to enhance the abundance of i*Bax* mRNA in EVs when i*Bax* was overexpressed in the donor cells. However, there was a marginal decrease in the i*Bax* mRNA level in the parental cells (Figures 4B and 4C).

Next, we investigated whether i*Bax* mRNA loading and surface functionalization by PTGFRN-MCP affected the size and morphology of modified EVs. Our results indicated that i*Bax* mRNA loading and PTGFRN-MCP functionalization did not significantly change the marker expression, morphology, or size compared to the unmodified control EVs (Figure 4D-F).

### Apoptosis induction by EV^iTx^ in senescent cells *in vitro*

Notably, *Bax* transfection alone did not affect cell apoptosis (Figure S5). BTSA1 is a BAX activator that converts dormant cytosolic BAX monomers into activated conformers that are transferred to the mitochondrial membrane 38. Delivering i*Bax* mRNA and BTSA1 to senescent cells may represent a novel approach for clearing senescent cells. Therefore, we constructed EV^iTx^ to deliver functional i*Bax* mRNA and BTSA1 into senescent cells. Briefly, HEK293T cells were co-transfected with the i*Bax* vector, PTGFRN-MCP vector, and TfR vector to obtain engineered i*Bax*@EV^TfR^, which were incubated with SMN-Tf to create i*Bax*@EV^SMN^. BTSA1 was then membrane-loaded onto i*Bax*@EV^SMN^ by incubation, resulting in EV^iTx^ (Figure S6A). EV^iTx^ represented typical EVs, with the dark SMN-Tf spots surrounding the spherical vesicles (Figure S6B). Western blot analysis revealed that EV^iTx^ did not alter the marker expression, while qPCR analysis showed a significant increase in *Bax* mRNA abundance in EV^iTx^ compared to EV^Ctrl^ (Figures S6C and S6D).

**Figure 5:**
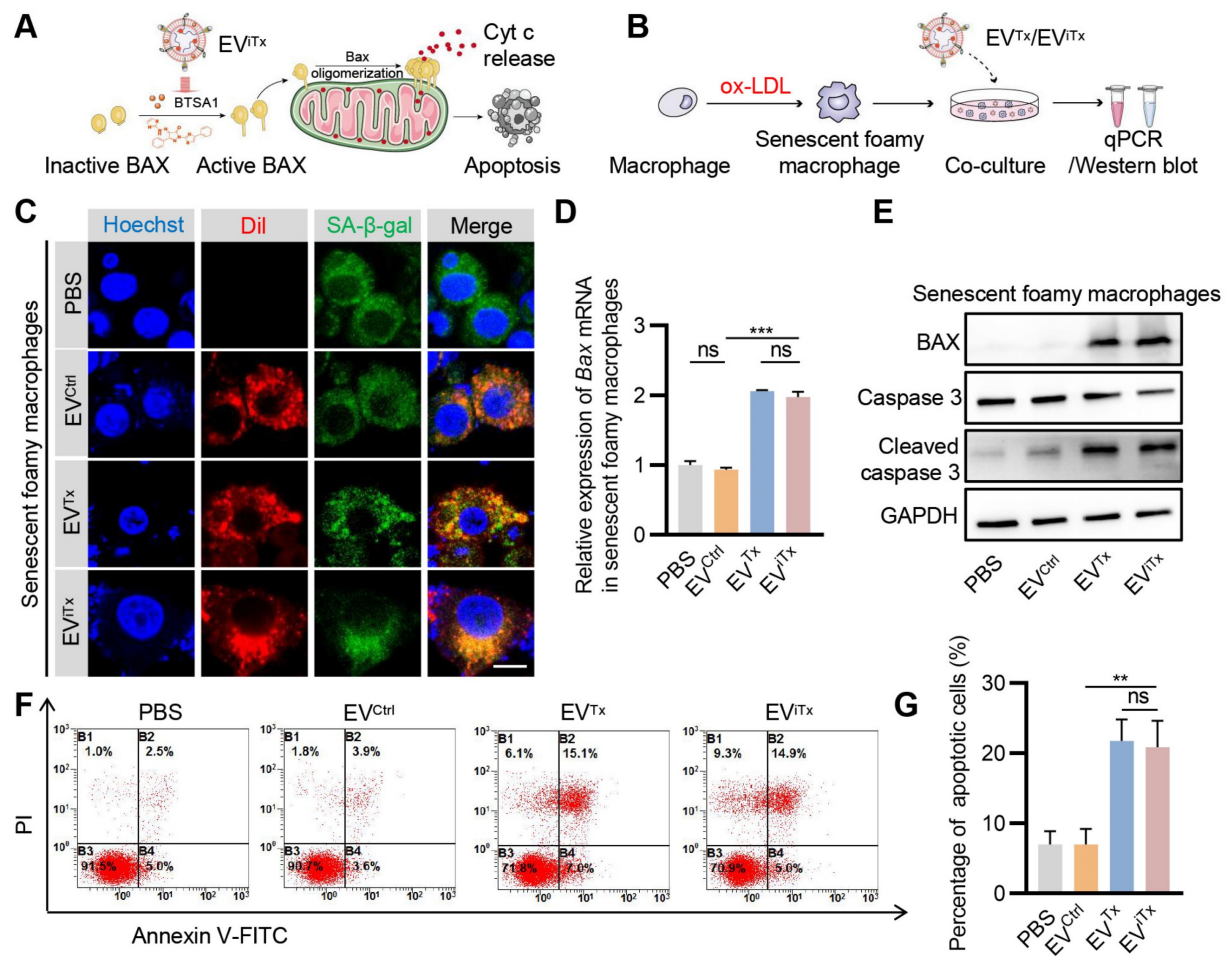
** Apoptosis induction by EV^iTx^ in senescent cells *in vitro*.** (A) Schematic illustrating the mechanism of EV^iTx^-mediated apoptosis of senescent foamy macrophages. (B) Schematic of EVs co-cultured with senescent foamy macrophages. (C) Uptake of EVs by senescent foamy macrophages. The intracellular localization of DiI-labeled EVs (in red) was assessed using fluorescence microscopy, while SA-β-gal was stained in green, and nuclei were counterstained in blue. Scale bar = 10 μm. (D) qPCR analysis of *Bax* mRNA in senescent foamy macrophages. (E) Western blot analysis to evaluate BAX or cleaved caspase-3 protein expression in senescent foamy macrophages treated with various EVs. (F) Flow cytometry detection of apoptotic senescent foamy macrophages. (G) Quantitative analysis of Figure 5F. Data are presented as mean ± SEM of three independent experiments. Statistical significance was determined by one-way ANOVA with Tukey's post hoc test. **p < 0.01. ***p < 0.001. ns, no significance.

Subsequently, we explored whether EV^iTx^ differentially induced apoptosis in healthy and senescent cells *in vitro* (Figure 5A). Senescent foamy macrophages were induced by ox-LDL treatment and verified by SA-β-gal activity and qPCR analysis of the senescent cell marker *p21* (Figure S7A-B). Although senescent foamy macrophages take up EV^Ctrl^, EV^Tx^, and EV^iTx^, only the EV^Tx^- and EV^iTx^-treated groups showed a significant increase in *Bax* mRNA expression compared to the control group (Figures 5B-D). BAX protein expression was also significantly increased following treatment with EV^Tx^ or EV^iTx^. Also, cleaved caspase-3 was observed in senescent foamy macrophages co-cultured with EV^Tx^ or EV^iTx^ (Figure 5E). Flow cytometry analysis revealed a significant increase in the percentage of apoptotic cells in the EV^Tx^- or EV^iTx^-treated group (Figures 5F and 5G). The results from the CCK-8 assay also demonstrated a gradual decrease in cell viability in the EV^Tx^ or EV^iTx^ treatment groups with increasing co-incubation time. At 24 h, the cell viability in these groups was less than 40% (Figures S8A-C).

Similarly, EV^Ctrl^, EV^Tx^, and EV^iTx^ were also taken up by healthy macrophages (Figures S9A-C). However, BAX protein was increased following EV^Tx^ or EV^iTx^ treatment, but no significant cleaved caspase-3 was observed (Figure S9D). Similar to the cleaved caspase-3 expression, no significant increase in the proportion of apoptotic cells was observed in the EV^Tx^- or EV^iTx^-treated groups (Figures S9E and S9F). The CCK-8 experimental results also demonstrated a marginal decline in cell viability in the EV^Tx^ or EV^iTx^ treatment groups with prolonged co-incubation time. As observed at the 24-h time point, the cell viability also remained above 80% (Figures S10A-C). In summary, our data showed that EV^Tx^ or EV^iTx^ induced apoptosis in senescent foamy macrophages while showing no significant effect on non-senescent macrophages, which could be explained by the higher sensitivity of senescent cells to the senolytics.

### Absence of significant apoptosis induction by EV^iTx^ in Huh7 cells

**Figure 6:**
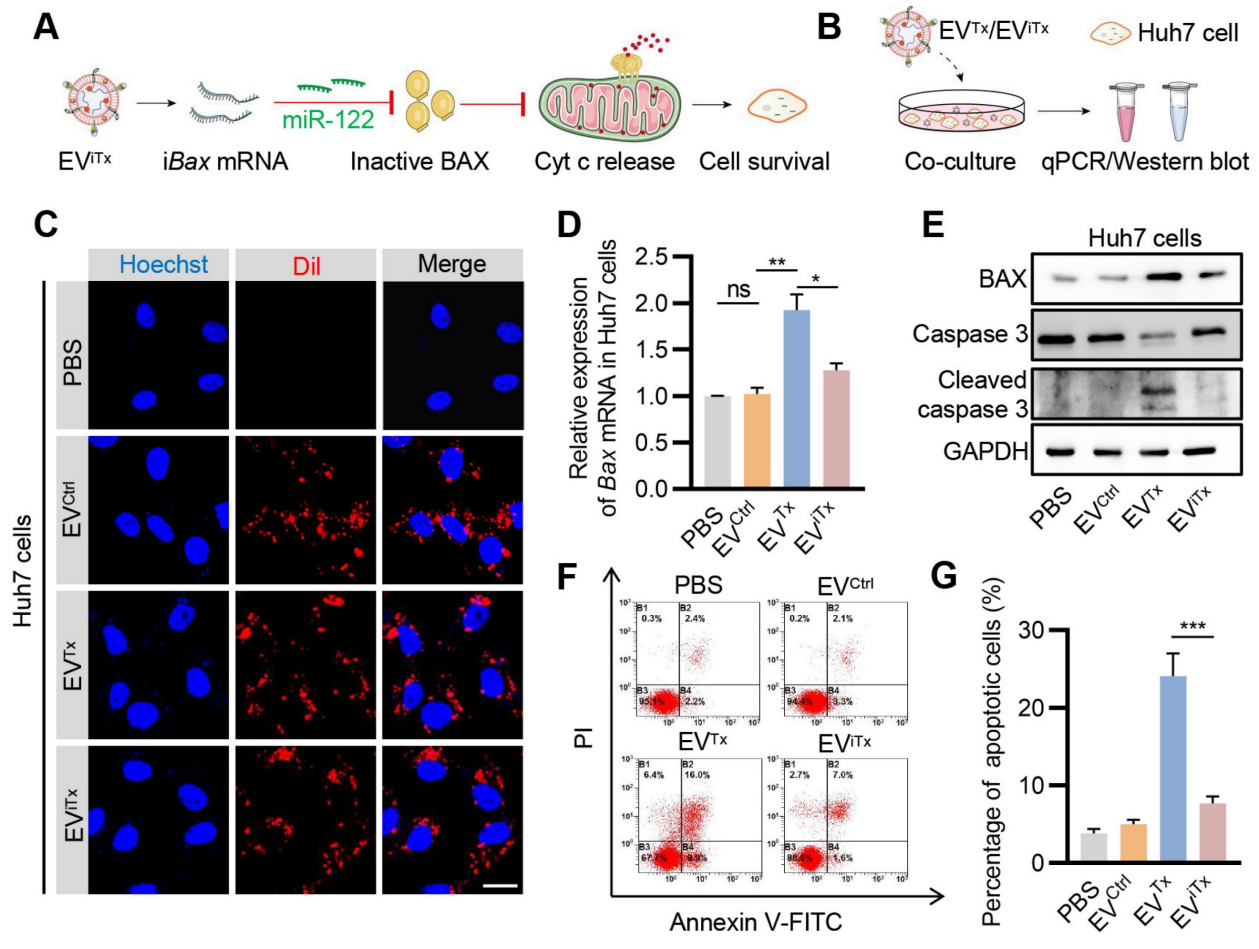
**Absence of significant apoptosis induction by EV^iTx^ in Huh7 cells.** (A) Schematic illustrates the mechanism by which Huh7 cells were treated with EV^iTx^ without undergoing apoptosis *in vitro*. (B) Schematic of EVs co-cultured with Huh7 cells. (C) Uptake of EVs by Huh7 cells. The intracellular localization of DiI-labeled EVs (in red) was assessed using fluorescence microscopy, while nuclei were counterstained with Hoechst (in blue). Scale bar = 20 μm. (D) qPCR analysis of *Bax* mRNA in Huh7 cells. (E) Western blot analysis to evaluate BAX or cleaved caspase-3 protein expression in Huh7 cells treated with various EVs. (F) Flow cytometry detection of apoptotic Huh7 cells. Data are presented as mean ± SEM of three independent experiments. (G) Quantitative analysis of Figure 6F. Statistical significance was determined by one-way ANOVA with Tukey's post hoc test. *p < 0.05. **p < 0.01. ***p < 0.001. ns, no significance.

Since EVs are inevitably accumulated in liver cells, we next asked whether EV^iTx^ induced apoptosis in liver cells by testing the effects of EV^Tx^ and EV^iTx^ on Huh7 cells (Figure 6A). First, the EV^iTx^ and other control EVs were DiI-labeled and then co-incubated with Huh7 cells (Figure 6B). While EV^Ctrl^, EV^Tx^, and EV^iTx^ were all taken up by Huh7 cells, only the EV^Tx^-treated group showed a significant increase in *Bax* mRNA and protein expression compared to the control groups (Figures 6C-E). Unlike healthy macrophages, the EV^Tx^-treated group demonstrated robust apoptosis, which was evident by increased expression of cleaved caspase-3 and flow cytometry (Figures 6E-G). The differences could be explained by the relatively low lysosomal activity of Huh7 cells and thus efficient delivery 39, 40. Notably, in contrast to the EV^Tx^-treated group, the EV^iTx^-treated group had no apparent apoptotic cells (Figures 6F and 6G). Collectively, these data suggested that EV^iTx^ is a safe senolytic strategy, preferentially killing senescent cells.

### Efficient attenuation of AS by EV^iTx^ in ApoE^-/-^ mice

**Figure 7:**
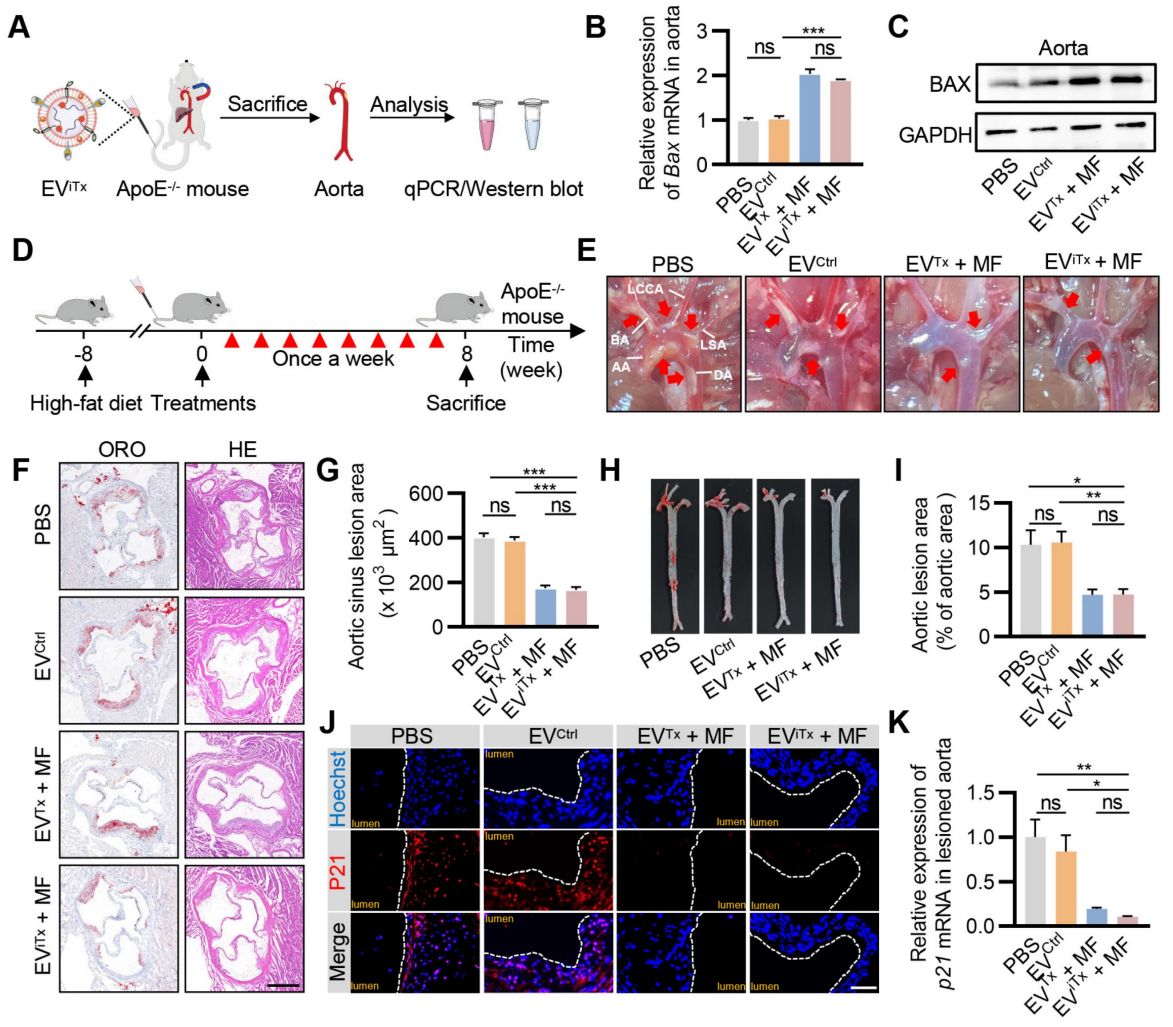
** Efficient attenuation of AS by EV^iTx^ in ApoE^-/-^ mice.** (A) ApoE^-/-^ mice were administered with EV^iTx^ *via* tail vein injection, while MF was applied to the aorta for 1 h. The aorta was then isolated for qPCR and Western blot analysis. (B) qPCR analysis of *Bax* mRNA levels in lesioned aorta. (C) Western blot analysis of BAX protein expression in lesioned aorta treated with various EVs. (D) Schematic of *in vivo* experimental procedures for AS in high-fat diet ApoE^-/-^ mice. (E) Representative aortic arch view of the atherosclerotic lesions in ApoE^-/-^ mice from indicated groups. (F) Representative images of aortic sinus cross-sections stained with ORO and H&E from ApoE^-/-^ mice in the indicated groups. Scale bar = 200 μm. (G) Statistical data of the ORO-positive plaque area from Figure 7F. (H) Representative images of ORO staining of the entire aorta in ApoE^-/-^ mice treated as above. (I) Percentage analysis of the atherosclerotic region from Figure 7H. (J) Immunofluorescence staining of P21 expression in aortic roots from high-fat diet ApoE^-/-^ mice in the indicated groups. Scale bar = 50 μm. (K) qPCR analysis of *p21* mRNA in aortic roots. AA, Ascending aorta; DA, Descending aorta; BA, Brachiocephalic artery; LCCA, left common carotid artery; LSA, left subclavian artery. Data are presented as mean ± SEM. n = 6 per group. Statistical significance was determined by one-way ANOVA with Tukey's post hoc test. *p < 0.05. **p < 0.01. ***p < 0.001. ns, no significance.

As there was a significant accumulation of senescent cells in atherosclerotic plaques (Figure S11), their targeted elimination in plaques may be a promising therapeutic strategy for treating AS. We first verified the ability of EV^iTx^ to deliver i*Bax* mRNA to plaque sites. ApoE^-/-^ mice were administered with EV^iTx^ *via* tail vein injection together with the application of MF to the aorta for 1 h. The aorta was then isolated for qPCR and Western blot analysis (Figure 7A). The results indicated a significant increase in *Bax* mRNA and protein expression in the aorta of mice treated with EV^Tx^ and EV^iTx^ (Figures 7B and 7C). A systematic analysis of the therapeutic effects of EV^iTx^ was subsequently conducted. Specifically, we fed ApoE^-/-^ mice a high-fat diet for 8 weeks, followed by weekly injections of the indicated EVs for another 8 weeks (Figure 7D). Gross inspection of the aorta indicated that mice treated with EV^Tx^ + MF and EV^iTx^ + MF had fewer atherosclerotic plaques than the control group, which received PBS or EV^Ctrl^ treatments (Figure 7E). Additionally, a cross-sectional view of Oil Red O (ORO) and hematoxylin-eosin (H&E) staining of the aortic roots revealed that the burden of atherosclerotic plaques, especially the lipid core, was significantly less in the EV^Tx^ + MF and EV^iTx^ + MF group than the control group (Figures 7F and 7G). These findings were further confirmed by ORO staining of the aortic tree (Figures 7H and 7I).

We also evaluated the population of senescent cells in plaques following treatment with EV^Tx^ + MF and EV^iTx^ + MF by P21 immunostaining. The control group exhibited a high number of P21^+^ cells in the plaques, whereas the infiltration of senescent cells was significantly reduced in the EV^Tx^ + MF- and EV^iTx^ + MF-treated groups (Figure 7J). This was further demonstrated by qPCR analysis from the aorta (Figure 7K). Furthermore, the expression of cell cycle regulator *p16^Ink4a^* and SASP factors, such as *Tnfα*, *Il1α*, *Mmp3*, and *Mmp13*, in the aortas were considerably suppressed in mice treated with EV^Tx^ + MF and EV^iTx^ + MF compared to the control group receiving PBS or EV^Ctrl^ treatments, as quantified by qPCR (Figures S12A-E). Collectively, these results demonstrated the potential of EV^iTx^ as a targeted senolytic for effective intervention against AS.

### Minimal side-effects of EV^iTx^ *in vivo*

**Figure 8:**
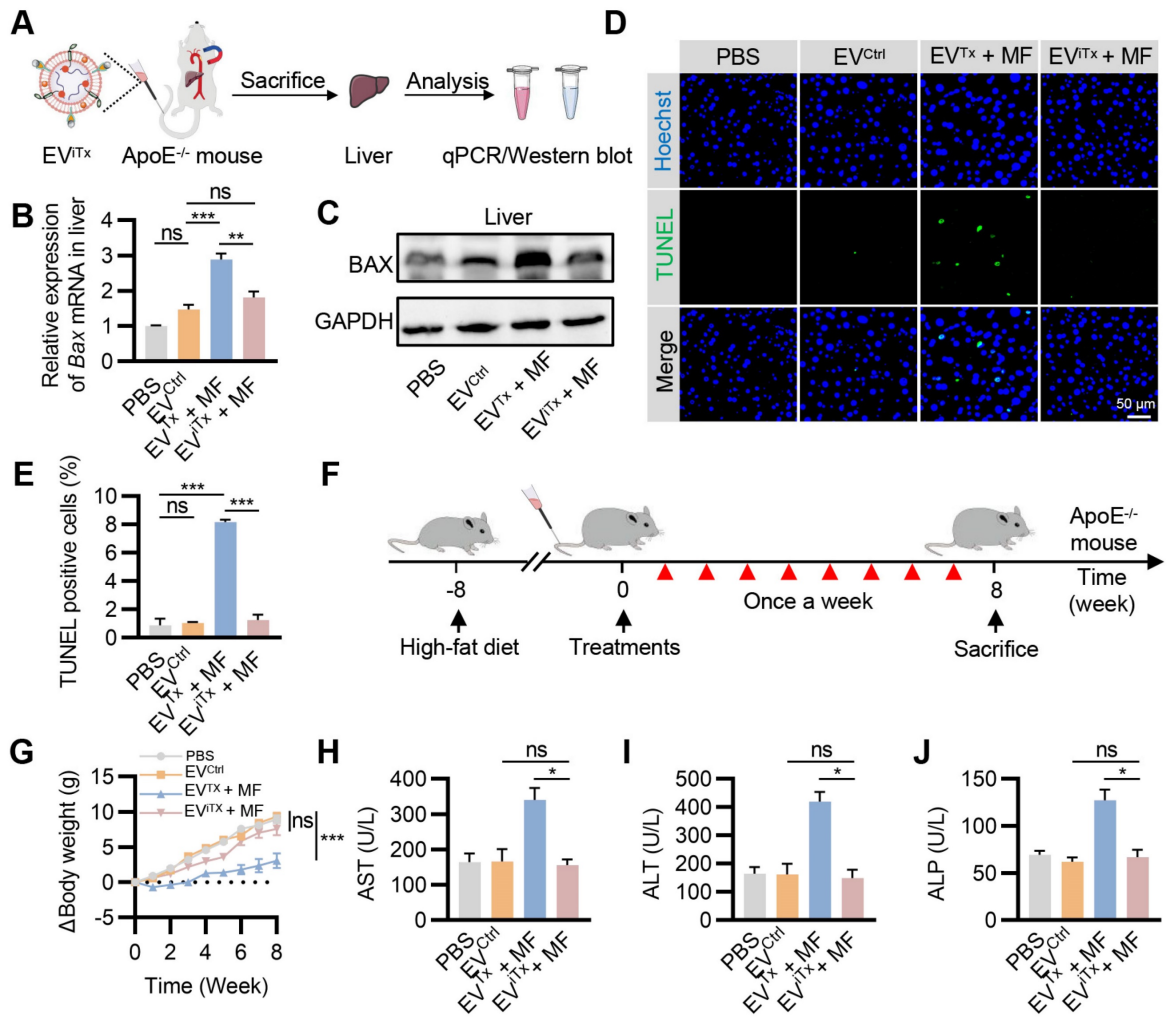
** Minimal side-effects of EV^iTx^ *in vivo*.** (A) ApoE^-/-^ mice were administered with EV^iTx^ *via* tail vein injection, while MF was applied to the aorta for 1 h. The liver was isolated for qPCR and Western blot analysis. (B) qPCR analysis of *Bax* mRNA in the liver in the indicated groups. (C) Western blot analysis of BAX protein expression in the liver in the indicated groups. (D) Representative images of the TUNEL staining of the liver from the ApoE^-/-^ mice with indicated treatments. (E) Quantitative analysis of Figure 8D. (F) Schematic of *in vivo* experimental procedures for AS in high-fat diet ApoE^-/-^ mice. (G) Body weight change curve in ApoE^-/-^ mice with indicated treatments. (H-J) Examination of the AST (H), ALT (I) and ALP (J) in ApoE^-/-^ mice treated as indicated. AST, Aspartate aminotransferase; ALT, Alanine aminotransferase; ALP, Alkaline phosphatase. Data are presented as mean ± SEM. n = 6 per group. Statistical significance was determined by one-way ANOVA with Tukey's post hoc test. *p < 0.05. **p < 0.01. ***p < 0.001. ns, no significance.

Due to the inherent *in vivo* distribution pattern of EVs, there was a preferential accumulation of EV^Tx^ and EV^iTx^ in the liver. We investigated whether i*Bax* mRNA delivered into the liver by EV^iTx^ could be effectively degraded. ApoE^-/-^ mice were administered with EV^iTx^ *via* tail vein injection, while MF was applied to the aorta for 1 h. The liver tissue was then isolated for qPCR and Western blot analysis (Figure 8A).

A significant decrease in *Bax* mRNA levels in the EV^iTx^ + MF group compared to the EV^Tx^ + MF group was detected by qPCR, but no significant differences were observed compared to the PBS control and EV^Ctrl^ groups (Figure 8B). Also, Western blot results showed trends similar to qPCR analysis (Figure 8C). Terminal deoxynucleotidyl transferase-mediated dUTP nick end labeling (TUNEL) staining of the liver revealed a significant increase in TUNEL-positive cells only in the EV^Tx^ + MF group. In contrast, compared to the control group, the EV^iTx^ + MF group showed no significant signs of apoptosis (Figures 8D and 8E). These data suggested that compared to EV^Tx^, EV^iTx^ could significantly reduce liver cell apoptosis caused by BAX overload. Subsequently, TUNEL staining was performed in other organs, including the heart, spleen, lungs, and kidneys. A small number of apoptosis-positive cells were observed in the lung and spleen after treatment with EV^Tx^ + MF or EV^iTx^ + MF (Figure S13), indicating minor toxicity to lung and spleen tissues, a limitation of our engineered EVs. Based on this result, a comprehensive *in vivo* assessment of the biocompatibility of EV^iTx^ was conducted.

Identical mouse models and dosing protocols were utilized to ensure consistency in the therapeutic trials *in vivo* (Figure 8F). During this period, the body weight of mice was monitored continuously. The results showed that mice in the EV^Tx^ + MF group had a slow weight gain of approximately 3 g, whereas mice in the EV^iTx^ + MF group had a weight gain trend similar to the control group, with an approximate 7.6 g weight gain (Figure 8G). The levels of Prostaglandin E2 (PGE2) and Thromboxane B2 (TXB2) in the plasma of mice were assessed using an enzyme-linked immunosorbent assay (ELISA). Our findings indicated no significant differences in PGE2 and TXB2 plasma levels between the EV^iTx^ + MF treatment and control groups (Figure S14). These data suggested that the EV treatment did not alter the profile of circulating monocytes and other white blood cells, and platelet activation and/or function. Blood biochemistry was performed to assess liver and kidney functions in each group. The results showed no statistically significant differences in liver function indicators (GGT, TBIL, DBIL, ALB, and TBA) and kidney function indicators (BUN and CREA) among the four groups (Figures S15 and S16). Notably, significantly higher levels of AST, ALT, and ALP were observed in the EV^Tx^ + MF group than in the control group, while no significant difference was found between the EV^iTx^ + MF and control groups (Figures 8H-J). Furthermore, no significant necrosis was observed by H&E staining of EV^Tx^-treated mice tissues. However, severe steatosis was observed, which could be attributed to the potential hepatotoxicity of EV^Tx^ treatment (Figure S17). Echocardiographic results demonstrated no significant differences in cardiac systolic and diastolic function in mice across all groups (Figure S18A-D). In summary, these data supported a much higher safety of EV^iTx^ over EV^Tx^.

## Discussion

In this study, a therapeutic EV (EV^iTx^) was engineered with SMN conjugated on the surface, i*Bax* mRNA encapsulated inside, and BAX activator BTSA1 incorporated into the membrane. Under external MF navigation, EV^iTx^ was delivered into atherosclerotic plaques and induced significant apoptosis in senescent cells regardless of origin. Notably, in liver cells, i*Bax* mRNA was translationally repressed by miR-122 endogenously expressed in liver cells and thus had minimal hepatotoxicity. Repeated delivery of EV^iTx^ *via* tail vein injection achieved high therapeutic efficacy and low side effects in ApoE^-/-^ mice.

Lipid-lowering therapy and anti-inflammatory strategies are the mainstream treatment paradigm for AS 41. Lipid-lowering measures focus predominantly on preventative methods, with long-term blood lipid management substantially reducing the risk of AS 42. However, these strategies favor early intervention but have limited efficacy in managing mature atherosclerotic plaques. While anti-inflammatory therapies exhibit considerable promise in arresting the progression of atherosclerosis, the results have been inconsistent, as illustrated by the divergent outcomes reported in the CANTOS and CIRT clinical trials 43, 44. Consequently, reliable therapeutic interventions for AS remain elusive.

Emerging evidence has highlighted the critical role of accumulating senescent cells and their SASP secretion in the plaques as a primary driver in the evolution and development of AS 45. Numerous studies have demonstrated that the systemic elimination of senescent cells could significantly attenuate AS 46, 47. However, the translation of this strategy into clinical practice has been impeded by the concomitant severe adverse side effects. However, the prospect of senescent cell clearance remains a promising therapeutic avenue for AS treatment. Our current study aimed to devise an innovative approach for targeting and eliminating senescent cells in plaque regions.

Currently, the clearance of senescent cells by senolytics is primarily achieved by inhibiting pro-survival pathways, such as BCL-2/BCL-XL, P53/P21, and PI3K/AKT signaling (“inhibit the inhibitors”) 13-16. Nonetheless, given the heterogeneity of senescent cells, their response to therapeutic targeting of various BCL-2 family members may differ 15. Besides, the senolytics, such as D, Q, and N, have limited ability to target specific senescent cell types 17. A combination of single-target drugs or a drug with multiple pro-survival targets may be necessary to improve the effectiveness of cellular clearance 13, 48. However, the combination approach resulted in significant side effects, including thrombocytopenia and neutropenia 18. The protein BAX is critical in initiating programmed cell death or apoptosis 19. The proposed strategies of “inhibit the inhibitor” have the potential to ultimately trigger the activation of apoptosis by inducing BAX oligomerization on the mitochondrial membrane 21.

In contrast to the prevailing methodologies, our study employed an innovative method of directly delivering *Bax* mRNA and activator (BTSA1) into senescent cells. This approach aimed to kill senescent cells by promoting their programmed elimination through an “activate the activator” strategy rather than suppressing their survival mechanisms. It could directly activate apoptosis independent of the anti-apoptotic mechanisms in the senescent cells and may have broad applicability across different senescent cell types. The proposed strategy to trigger cell death in healthy, non-senescent cells was also avoided. Compelling evidence indicated that senescent cells are “primed for death”, due to significantly lower levels of anti-apoptotic reserves than normal cells 14. Low-dose activation of BAX has been shown to effectively initiate apoptosis in senescent cells. Conversely, non-senescent cells maintain sufficient anti-apoptotic reserves, counteracting low-dose activated BAX through pro-survival proteins such as BCL-2 and BCL-XL that allow them to survive.

Due to their low immunogenicity and superior biocompatibility, EVs have been identified as efficacious carriers for co-delivering *Bax* mRNA and BTSA1 to senescent cells. Nonetheless, the accurate identification and clearance of senescent cells within atherosclerotic plaques necessitate precisely targeting this diverse cell population. Strategies such as targeted peptide modification, contingent on specific ligand-receptor binding, are commonly employed in targeted delivery systems 49. However, senescent cells within atherosclerotic plaques exhibit considerable heterogeneity and are not a homogeneous cell type with consistent cellular origin. Given the lack of universal surface markers for senescent cells, targeted peptide delivery systems are insufficient for effectively clearing senescent cells within plaques. Thus, accurate targeting of this specific cell population remains a complex task. The present study employed SMN-modified EVs as delivery vehicles to accurately identify different types of senescent cells in the plaque region. This unique targeting ability of EV^SMN^ was not dependent on the cell type but rather on the recognition of the spatial location of the affected tissue, rendering it a suitable method for delivering drugs to senescent cells in plaques. Our data indicated that EV^SMN^ accumulated preferentially in the plaque region with the assistance of MF and co-localized with P21^+^ cells, potentially including senescent foamy cells or other types of senescent cells. Our observations suggested that magnetically-guided delivery of EV^iTx^ to plaques might have clinical applications.

Generally, nanoparticles, including the EVs, are accumulated in the liver, resulting in liver injury when *Bax* mRNA is excessively delivered 29, 30. Therefore, repressing *Bax* translation in liver cells is imperative to avoid potential toxicity. MicroRNAs play a crucial role in gene expression regulation by destabilizing target mRNAs or inhibiting their translation 31. miR-122, a mammalian liver-specific non-coding polyadenylated RNA with an average count of 50,000-82,000 copies per cell in the adult liver 32, 33, could serve as a potential candidate for repressing i*Bax* mRNA to circumvent BAX-related hepatotoxicity. For constructing the miR-122-repressed i*Bax* mRNA expression system, three sequences complementary to miR-122 were inserted for cloning the expression plasmid. Thus, although EV^iTx^ inevitably accumulated in liver cells, i*Bax* mRNA was translationally repressed by miR-122, thus protecting liver cells from the potential toxicity of BAX.

Our study has some inherent limitations. First, despite magnetic targeting to enhance the accumulation of EV^iTx^ at plaque sites in the aorta and employing engineered i*Bax* mRNA to address hepatotoxicity, a slight degree of injury was observed in the lungs and spleen due to the buildup of i*Bax* mRNA. To circumvent these adverse effects, future improvement strategies should focus on using tissue-specific miRNAs and integrating their recognition sites into the 3'-UTR of the i*Bax* vector to mitigate such side effects. Second, EV^iTx^ treatment led to increased level of apoptosis compared to the basal condition, suggesting that further improvement to avoid the side-effects is still needed. Finally, due to the ease of modification of HEK293T cells, we selected EVs derived from these cells as delivery vehicles. However, the cancer origin of HEK293T cells as a source of EVs is not a viable delivery platform, imposing limitations on the translational potential of engineered EVs. Future work should explore mesenchymal stem cells as a promising alternative source of EVs for clinical translation.

## Conclusion

The current study utilized EV^iTx^ for the targeted delivery of i*Bax* mRNA and BTSA1 into senescent cells to directly induce apoptosis (“activate the activator”). Using ApoE^-/-^ mice, we demonstrated that the engineered EV^iTx^ effectively eliminated senescent cells in plaque regions and reduced SASP secretion, significantly attenuating the atherosclerotic burden in the aortas. Notably, no significant side effects were observed. Thus, the EV^iTx^-based strategy offers a promising treatment approach for AS and other age-related diseases.

## Materials and Methods

### Materials

The SMNs were procured from Nanjing Nanoeast Biotech Co., Ltd (Nanjing, China). The reagents EDAC, sulfo-NHS, Tf, and 2-mercaptoethanol were obtained from Sigma (St Louis, MO, USA). BTSA1 was purchased from Cell Signal Technology (Boston, USA). The Pierce BCA protein assay kit was purchased from Thermo Scientific (Waltham, Massachusetts, USA), and the Tripure Isolation Reagent was procured from Roche (Basel, Switzerland). The RIPA lysis buffer was obtained from Beyotime Biotechnology (Shanghai, China).

### Synthesis of SMN-Tf

SMN-Tf was synthesized using the previously reported method 26. Briefly, SMN (20 μL, 4 mg/mL) was mixed with EDAC and sulfo-NHS in a molar ratio of 1:2:3 (pH = 5.5) and incubated at ambient temperature for 1 h. The reaction was stopped by adding 1 μL of 2-mercaptoethanol. The activated SMN was separated magnetically and resuspended in 200 μL of borate buffer (20 mM, pH = 8.5). Subsequently, 10 μg of Tf was added to the solution, and the mixture was incubated for 12 h at 4 °C under nitrogen. Following magnetic separation, SMN-Tf was isolated, washed 3 times with PBS, resuspended in 200 μL PBS, and stored at 4 °C for subsequent experimental procedures.

### Synthesis of EV^iTx^

HEK293T cells were co-transfected with PTGFRN-MCP, i*Bax*, and TfR vectors to obtain engineered i*Bax*@EV^TfR^. Next, 100 μL of the i*Bax*@EV^TfR^ solution at a protein concentration of 1μg μL^-1^ was mixed with 100 μL of SMN-Tf solution and incubated for 4 h at 4 °C on a shaker. Subsequently, i*Bax*@EV^SMN^ was obtained by magnetic separation, resuspended in PBS, and 100 µg of purified i*Bax*@EV^SMN^ (0.5 µg µL^-1^) was incubated with 2 µL of BTSA1 (5 mM) for 1 h in an ice bath. Subsequently, i*Bax*@EV^SMN^ loaded with BTSA1 (EV^iTx^*)* was obtained by magnetic separation, washed, and resuspended in cold PBS for subsequent experiments.

### EV isolation and characterization

Cells were subjected to serum deprivation for 48 h, and the cultured medium was collected and centrifuged at 500 *g* for 10 minutes to remove cells, followed by centrifugation at 10,000 *g* for 20 minutes to eliminate residual cellular debris. After filtering through 0.22 μm filters, the resulting supernatant was subjected to ultracentrifugation at 100,000 *g* for 3 h for EV harvesting. The EV-containing pellet was washed, resuspended in sterile PBS, and stored at -80 °C for subsequent experimental procedures.

The protein concentration of EVs was determined by the BCA Protein Assay Kit. For biomarker analysis, the EV pellet was dissolved in RIPA lysis buffer for Western blot analysis. The size and morphology of EVs were determined using TEM (JEM1400, JEOL, Japan). The size distribution of EVs was analyzed by NTA using Nanosight (NS300, Malvern, UK).

### Cell culture and senescence induction

HEK293T and Huh7 cells were cultured in DMEM high glucose medium (HyClone, Logan, USA) supplemented with 10% fetal bovine serum (FBS) and 1% penicillin/streptomycin (HyClone, Logan, USA). RAW264.7 cells were cultured in RPMI-1640 medium (Gibco, Carlsbad, USA) supplemented with 1% L-glutamine, while the remaining components remained unchanged. Cells were maintained in a humidified incubator at 37°C with 5% CO_2_.

To establish an *in vitro* model of senescent foamy macrophages, RAW264.7 cells were selected as the cellular model and stimulated with ox-LDL. The cells (less than 10 passages) were cultured in the presence of 50 μg mL^-1^ ox-LDL for 24 h, followed by incubation in fresh medium for another day before conducting subsequent experiments 50.

### Plasmid construction

Synthesis and cloning of recombinant genes i*Bax*, *Bax*, and TfR fragment into the pcDNA3.1(+) plasmid and cloning the recombinant gene PTGFRN-MCP fragment into the pcDNA3.1(-) plasmid were performed by Genscript Biotech Corporation. The detailed sequences employed for the synthesis are presented in Table S1.

### Cell transfection

HEK293T and Huh7 cells were seeded in 6-well plates one day prior to transfection. The cells were transfected with *Bax*/i*Bax* plasmids or *Bax*/i*Bax* plasmids in combination with miR-122 mimics/inhibitors using HighGene Transfection reagent (ABclonal Technology, Wuhan, China) as per manufacturer's instructions. Six hours later, the medium was replaced with the complete medium containing 10% FBS. Sequences of miRNA mimics or inhibitors are displayed in Table S2.

### RNA isolation and real-time polymerase chain reaction

Total RNA was isolated from tissues, cultured cells, or EVs utilizing Tripure Isolation Reagent according to the manufacturer's protocol. Subsequently, mRNA was reverse transcribed using the First Strand cDNA Synthesis Kit (Genenode, Beijing, China) following the manufacturer's guidelines. The qPCR reactions were performed using FastStart Essential DNA Green Master (Roche, IN, USA) with specific primers. The primer sequences for qPCR are presented in Table S3. Target mRNA expression was normalized individually against GAPDH, and the relative expression of each target gene was calculated using the 2^-ΔΔCt^ method.

### Western blot analysis

The isolated tissues, cells, or EVs were treated with RIPA lysis buffer supplemented with the Protease Inhibitor Cocktail (Roche) at 4 °C for 30 minutes. The protein concentrations were assessed using the BCA Protein Assay Kit. Subsequently, equal amounts of purified proteins were separated using SDS-PAGE and transferred onto a nitrocellulose membrane in an ice bath. The nitrocellulose membrane was blocked with 5-8% nonfat dried milk for 1 h at room temperature, followed by overnight incubation with primary antibodies at 4 °C. The antibodies employed were mouse anti-CD81 (Abcam, ab79559), mouse anti-TSG101 (Abcam, ab83), rabbit anti-GM130 (Proteintech, 11308-1-AP), mouse anti-BAX (ProteinTech, 60267-1-Ig), rabbit anti-TfR (Proteintech, 10084-2-AP), rabbit anti-caspase-3 (Cell Signaling Technology, #14220), and mouse anti-GAPDH (Proteintech, 60004-1-Ig). The nitrocellulose membrane was subsequently incubated with corresponding secondary antibodies at room temperature for 1 h and visualized using the ECL Prime Western Blotting Detection Reagent (GE, UK).

### EV labeling

DiI and DiR, fluorescent probes widely used as lipophilic tracers for membrane labeling, were employed in this study for labeling EVs. Briefly, EVs (1 μg μL^-1^) were incubated with DiI or DiR dye (1 mM) at a ratio of 500:1 (volume) at 4 ℃ for 30 minutes. Unbound dyes were removed by centrifugation or magnetic separation.

### Cell viability assay

Cell viability was measured using a CCK-8 kit (Yeasen, China, 40203ES60). HEK293T cells were seeded in 96-well plates and transfected with various plasmids at 80% cell confluency using the protocol mentioned above. After 24 h, the CCK-8 solution was added, and absorbance was measured at 450 nm using a microplate reader (EPOCH, Bio-Tek) to determine cell viability.

RAW264.7 cells were seeded in 24-well plates and subjected to either ox-LDL stimulation or control conditions. After 24 h, the culture medium was refreshed, and the cells were co-incubated with EV^Tx^/EV^iTx^ at a final concentration of 100 µg mL^-1^. After 8, 16, or 24 h, the CCK-8 solution was added, and the absorbance at 450 nm was measured using a microplate reader to assess cell viability.

### Cell apoptosis analysis

Apoptosis efficiency of senescent foamy macrophages after EV^Tx^/EV^iTx^ treatments: RAW264.7 cells were seeded in 6-well plates and treated with ox-LDL (50 μg mL^-1^). After 24 h, the medium was replaced with fresh medium and co-incubated with EV^Tx^/EV^iTx^ at a final concentration of 100 µg mL^-1^. After 12 h, cells were detached using trypsin (without EDTA) and washed thrice with cold PBS. The cells were then stained using an Annexin V-FITC Apoptosis Detection Kit I (BD Pharmingen, 556547) and analyzed using a flow cytometer (Coulter-XL, Apoptosis Analysis Software: EXPO32 ADC Analysis).

Apoptosis efficiency of RAW264.7 or Huh7 cells after EV^Tx^/EV^iTx^ treatments: RAW264.7 or Huh7 cells were seeded in 6-well plates. After 24 h, the medium was replaced with fresh medium and co-incubated with EV^Tx^/EV^iTx^ at a final concentration of 100 µg mL^-1^. Following a 12-h incubation period, the cells were collected for analysis using a flow cytometer.

### EV tracking analysis

DiI-labeled EVs were incubated with healthy macrophages or Huh7 cells for 6 h to visualize the internalization and tracing of EVs *in vitro*. The cells were then washed thoroughly with PBS, fixed with 4% paraformaldehyde (PFA) for 10 minutes, and washed again with PBS. Cell nuclei were stained with Hoechst for 10 minutes at room temperature. Non-specific adhesions were removed by washing the cells with PBS at the end of the experiment. RAW264.7 cells were cultured in confocal dishes for generating senescent foamy macrophages and induced with ox-LDL (50 μg mL^-1^) for 24 h. Subsequently, DiI-labeled EVs were added and incubated for 6 h. The cells were then washed thoroughly with PBS, fixed with 4% PFA for 3 minutes, and stained for senescence-associated β-galactosidase (SA-β-gal) using the SPiDER-βGal cellular senescence detection kit (SPiDER-βGal, Dojindo). Cell nuclei were stained as described above. Imaging was performed using a Nikon A1 Spectral Confocal Microscope (Nikon).

We chose 8-week-old male ApoE^-/-^ mice maintained on a high-fat diet for 8 weeks to conduct fluorescent *in vivo* tracing of EVs. DiR-labeled EV^none^ or EV^SMN^ (200 μg) were injected *via* the tail vein while applying a MF (0.6 T) for 1 h and the mice were euthanized after 12 h. Vital organs (heart, liver, spleen, lung, kidney, aorta) were isolated, and the distribution of the EVs in different organs was imaged using the IVIS^®^ Lumina II *in vivo* imaging system (PerkinElmer, Thermo Fisher). As a control, the mice were also injected with PBS *via* the tail vein.

### Experimental animals

Eight-week-old male ApoE^-/-^ mice were procured from the Model Animal Research Center of Nanjing University. Following a week-long acclimatization period, the mice were fed a high-fat diet (D12492, Research Diet, comprising 45% kcal from fat, 20% kcal from protein, and 20% kcal from carbohydrates) for 8 weeks to induce atherosclerosis.

The efficiency of plaque delivery of EV^iTx^ carrying i*Bax* mRNA and its degradation in the liver in high-fat diet ApoE^-/-^ mice were evaluated. The mice were administered with EVs at a dose of 4 μg/g body weight *via* tail vein injection, while MF was applied to the aorta for 1 h. After three injections of EVs, all animals were subjected to euthanasia, and their aorta and liver were extracted for further analytical studies.

For EV intervention, the mice were administered with EVs (4 μg/g body weight) *via* the tail vein on a weekly basis over the course of 8 weeks. For each injection of EVs, MF was applied near the aorta of the mice for 1 h. At the culmination of the experiment, all animals were subjected to euthanasia, and their tissues and major organs were extracted for further analytical studies. All animal procedures strictly adhered to the guidelines established by the Animal Care and Use Committee of Fourth Military Medical University.

### Histology and TUNEL

The experimental mice were anesthetized and then sacrificed after heart perfusion. All major organs, including the aorta, heart, liver, spleen, lung, and kidney, were extracted and fixed in 4% PFA for 24 h, and the surrounding adipose tissue was removed. Subsequently, the specimens were transferred to PBS containing 30% sucrose overnight to remove excess water. The organ samples were then embedded in OCT and sliced into 5 µm sections. ORO staining and H&E staining were carried out according to the established protocols. The lipid core area and the size of lesions were computed using ImageJ software. The histological changes in the major organs were assessed by two independent experts based on the H&E-stained specimens.

For TUNEL staining, three representative slides were selected for each mouse, and the presence of apoptotic cells in the liver tissue was evaluated using a TUNEL apoptosis detection kit (Promega,USA). The percentage of apoptotic cells was calculated based on the ratio of green fluorescence-positive cells. The cell numbers were quantified using ImageJ software.

### ELISA

Blood samples were collected by extracting the eyeball to evaluate the levels of TXB2 and PGE2. The serum was subsequently collected and subjected to analysis for TXB2 and PGE2 levels using corresponding commercial ELISA kit (Elabscience, Wuhan, China). The assay was conducted following the manufacturer's instructions.

### Serum biochemistry

Following an 8-h fasting period, blood samples were obtained by extracting the eyeball to evaluate liver and kidney functions. Liver function was assessed by measuring alanine aminotransferase (ALT), aspartate aminotransferase (AST), albumin (ALB), alkaline phosphatase (ALP), gamma-glutamyl transferase (GGT), total bilirubin (TBIL), direct bilirubin (DBIL) and total bile acid (TBA), while kidney function was evaluated through the examination of blood urea nitrogen (BUN) and creatinine (CREA) levels.

### Echocardiography

Prior to echocardiography, mouse hair was removed from the chest to the abdomen using a chemical depilatory agent. The mice were then placed on a temperature-controlled heating pad and anesthetized with isoflurane. Echocardiography was performed on the mice by an experienced technician using a Vevo 2100 imaging system (FUJIFILM VisualSonics, Canada). Several cardiac function parameters were evaluated using echocardiography, including M mode echocardiography, mitral flow Doppler echocardiography, systolic function parameter LVEF, and diastolic function parameter E/A. The heart rate was maintained between 400-500 beats per minute during the examination. All evaluated parameters were averaged over 5 cardiac cycles.

### Statistical analysis

All data were presented as the mean ± standard error of the mean (SEM). Student's *t*-test was employed to compare two groups, while one-way ANOVA was used for more than three groups. Statistical significance was determined using GraphPad Prism 9.0, and *p* < 0.05 was considered significant. * *p* < 0.05, ** *p* < 0.01, ***p < 0.001.

## Supplementary Material

Supplementary figures and tables.
