# Supplementary material for: Targeted elimination of senescent cells by engineered extracellular vesicles attenuates atherosclerosis in ApoE-/- mice with minimal side effects
Source: Theranostics. 2023 Sep 18;13(14):5114–29. doi: 10.7150/thno.87484 (PMC10526664; doi:10.7150/thno.87484)
Supplement: Supplementary file 1 — Supplementary figures and tables. [file thnov13p5114s1.pdf]

Supplementary Data

Targeted elimination of senescent cells by engineered extracellular vesicles attenuates atherosclerosis with minimal side effects in ApoE$^{-/-}$ mice

Liang Zhang[1,#], Chen Wang[1,#], Wei Hu[1,#], Te Bu[1], Wenqi Sun[1], Tian Zhou[1], Shuo Qiu[1], Mengying Wei[2], Helin Xing[3], Zhelong Li[1,✉], Guodong Yang[2,✉], and Lijun Yuan[1,✉]

1. Department of Ultrasound Diagnostics, Tangdu Hospital, Fourth Military Medical University, Xi'an, People's Republic of China
2. State Key Laboratory of Holistic Integrative Management of Gastrointestinal Cancers, Department of Biochemistry and Molecular Biology, Fourth Military Medical University, Xi'an, People's Republic of China
3. Department of Prosthodontics, Beijing Stomatological Hospital and School of Stomatology, Capital Medical University, Beijing, 100050, China

[#] These authors contributed equally to this article.

✉ Corresponding authors:
Lijun Yuan, Department of Ultrasound Diagnostics, Tangdu Hospital, Fourth Military Medical University, Xinsi Road NO.569th, 710038, Xi'an, China, email: yuanlj@fmmu.edu.cn; Tel: +862984777471, Fax: +862984777471
Guodong Yang, The State Laboratory of Cancer Biology, Department of Biochemistry and Molecular Biology, Fourth Military Medical University, Changlexi Road NO.169th, 710032, Xi'an, email: yanggd@fmmu.edu.cn; Tel: +862984774516, Fax: +862984774516
Zhelong Li, Department of Ultrasound Diagnostics, Tangdu Hospital, Fourth Military Medical University, Xinsi Road NO.569th, 710038, Xi'an, China, email: lzlfmmu@foxmail.com; Tel: +862984777471, Fax: +862984777471

Table S1. Sequences of the plasmids used in this study.

Plasmid	Sequence
TfR	GAATTCGCCACCATGGATCAAGCTAGATCAGCATTCTCTAACTTGTTTGGTGGAGAAC<br>CATTGTCATATACCCGGTTCAGCCTGGCTCGGCAAGTAGATGGCGATAACAGTCATGT<br>GGAGATGAAACTTGCTGTAGATGAAGAAGAAAATGCTGACAATAACACAAAGGCCAA<br>TGTCACAAAACCAAAAAGGTGTAGTGGAAGTATCTGCTATGGGACTATTGCTGTGATC<br>GTCTTTTTCTTGATTGGATTTATGATTGGCTACTTGGGCTATTGTAAAGGGGTAGAACC<br>AAAAACTGAGTGTGAGAGACTGGCAGGAACCGAGTCTCCAGTGAGGGAGGAGCCA<br>GGAGAGGACTTCCCTGCAGCACGTCGCTTATATTGGGATGACCTGAAGAGAAAGTTG<br>TCGGAGAAACTGGACAGCACAGACTTCACCGGCACCATCAAGCTGCTGAATGAAAAT<br>TCATATGTCCCTCGTGAGGCTGGATCTCAAAAAGATGAAAATCTTGCGTTGTATGTTG<br>AAAATCAATTTCGTGAATTTAAACTCAGCAAAGTCTGGCGTGATCAACATTTTGTTAA<br>GATTCAGGTCAAAGACAGCGCTCAAAACTCGGTGATCATAGTTGATAAGAACGGTAG<br>ACTTGTTTACCTGGTGGAGAATCCTGGGGGTTATGTGGCGTATAGTAAGGCTGCAACA<br>GTTACTGGTAAACTGGTCCATGCTAATTTTGGTACTAAAAAAGATTTTGAGGATTTATA<br>CACTCCTGTGAATGGATCTATAGTGATTGTCAGAGCAGGGAAAATCACCTTTGCAGAA<br>AAGGTTGCAAATGCTGAAAGCTTAAATGCAATTGGTGTGTTGATATACATGGACCAGA<br>CTAAATTTCCCATTGTTAACGCAGAACTTTCATTCTTTGGACATGCTCATCTGGGGACA<br>GGTGACCCTTACACACCTGGATTCCCTTCCTTCAATCACACTCAGTTTCCACCATCTCG<br>GTCATCAGGATTGCCTAATATACCTGTCCAGACAATCTCCAGAGCTGCTGCAGAAAAG<br>CTGTTTGGGAATATGGAAGGAGACTGTCCCTCTGACTGGAAAACAGACTCTACATGTA<br>GGATGGTAACCTCAGAAAGCAAGAATGTGAAGCTCACTGTGAGCAATGTGCTGAAAG<br>AGATAAAAATTCTTAACATCTTTGGAGTTATTAAAGGCTTTGTAGAACCAGATCACTAT<br>GTTGTAGTTGGGGCCCAGAGAGATGCATGGGGCCCTGGAGCTGCAAAATCCGGTGTA<br>GGCACAGCTCTCCTATTGAAACTTGCCCAGATGTTCTCAGATATGGTCTTAAAAGATG<br>GGTTTCAGCCCAGCAGAAGCATTATCTTTGCCAGTTGGAGTGCTGGAGACTTTGGATC<br>GGTTGGTGCCACTGAATGGCTAGAGGGATACCTTTCGTCCCTGCATTTAAAGGCTTTC<br>ACTTATATTAATCTGGATAAAGCGGTTCTTGGTACCAGCAACTTCAAGGTTTCTGCCAG<br>CCCACTGTTGTATACGCTTATTGAGAAAACAATGCAAAATGTGAAGCATCCGGTTACT<br>GGGCAATTTCTATATCAGGACAGCAACTGGGCCAGCAAAGTTGAGAAACTCACTTTA<br>GACAATGCTGCTTTCCCTTTCCTTGCATATTCTGGAATCCCAGCAGTTTCTTTCTGTTTT<br>TGCGAGGACACAGATTATCCTTATTTGGGTACCACCATGGACACCTATAAGGAACTGA<br>TTGAGAGGATTCCTGAGTTGAACAAAGTGGCACGAGCAGCTGCAGAGGTCGCTGGT<br>CAGTTCGTGATTAAACTAACCCATGATGTTGAATTGAACCTGGACTATGAGAGGTACA<br>ACAGCCAACTGCTTTCATTTGTGAGGGATCTGAACCAATACAGAGCAGACATAAAGG<br>AAATGGGCCTGAGTTTACAGTGGCTGTATTCTGCTCGTGGAGACTTCTTCCGTGCTAC<br>TTCCAGACTAACAACAGATTTCGGGAATGCTGAGAAAACAGACAGATTTGTCATGAA<br>GAAACTCAATGATCGTGTCATGAGAGTGGAGTATCACTTCCTCTCTCCCTACGTATCTC<br>CAAAAGAGTCTCCTTTCCGACATGTCTTCTGGGGCTCCGGCTCTCACACGCTGCCAGC<br>TTTACTGGAGAACTTGAAACTGCGTAAACAAAATAACGGTGCTTTTAATGAAACGCTG<br>TTCAGAAACCAGTTGGCTCTAGCTACTTGGACTATTCAGGGAGCTGCAAATGCCCTCT<br>CTGGTGACGTTTGGGACATTGACAATGAGTTTTAACTCGAG
i*Bax*	GAATTCGCCACCATGGACGGGTCCGGGGAGCAGCTTGGGAGCGGCGGGCCCACCAG<br>CTCTGAACAGATCATGAAGACAGGGGCCTTTTTGCTACAGGGTTTCATCCAGGATCGA<br>GCAGGGAGGATGGCTGGGGAGACACCTGAGCTGACCTTGGAGCAGCCGCCCCAGGA<br>TGCGTCCACCAAGAAGCTGAGCGAGTGTCTCCGGCGAATTGGAGATGAACTGGACA<br>GCAATATGGAGCTGCAGAGGATGATTGCTGACGTGGACACGGACTCCCCCCGAGAGG

	TCTTCTTCCGGGTGGCAGCTGACATGTTTGCTGATGGCAACTTCAACTGGGGCCGCGT<br>GGTTGCCCTCTTCTACTTTGCTAGCAAACTGGTGCTCAAGGCCCTGTGCACTAAAGTG<br>CCCGAGCTGATCAGAACCATCATGGGCTGGACACTGGACTTCCTCCGTGAGCGGCTG<br>CTTGTCTGGATCCAAGACCAGGGTGGCTGGGAAGGCCTCCTCTCCTACTTCGGGACC<br>CCCACATGGCAGACAGTGACCATCTTTGTGGCTGGAGTCCTCACCGCCTCGCTCACCA<br>TCTGGAAGAAGATGGGCTGAAATTCAAACACCATTGTCACACTCCAAATTCAAACAC<br>CATTGTCACACTCCAAATTCAAACACCATTGTCACACTCCAAATTACATGAGGATCAC<br>CCATGTACATGAGGATCACCCATGTCTCGAG
Bax	GAATTCGCCACCATGGACGGGTCCGGGGAGCAGCTTGGGAGCGGCGGGCCCACCAG<br>CTCTGAACAGATCATGAAGACAGGGGCCTTTTTGCTACAGGGTTTCATCCAGGATCGA<br>GCAGGGAGGATGGCTGGGGAGACACCTGAGCTGACCTTGGAGCAGCCGCCCCAGGA<br>TGCGTCCACCAAGAAGCTGAGCGAGTGTCTCCGGCGAATTGGAGATGAACTGGACA<br>GCAATATGGAGCTGCAGAGGATGATTGCTGACGTGGACACGGACTCCCCCCGAGAGG<br>TCTTCTTCCGGGTGGCAGCTGACATGTTTGCTGATGGCAACTTCAACTGGGGCCGCGT<br>GGTTGCCCTCTTCTACTTTGCTAGCAAACTGGTGCTCAAGGCCCTGTGCACTAAAGTG<br>CCCGAGCTGATCAGAACCATCATGGGCTGGACACTGGACTTCCTCCGTGAGCGGCTG<br>CTTGTCTGGATCCAAGACCAGGGTGGCTGGGAAGGCCTCCTCTCCTACTTCGGGACC<br>CCCACATGGCAGACAGTGACCATCTTTGTGGCTGGAGTCCTCACCGCCTCGCTCACCA<br>TCTGGAAGAAGATGGGCTGAACATGAGGATCACCCATGTACATGAGGATCACCCATGT<br>CTCGAG
PTGFRN-MCP	GCTAGCGCCACCATGGGGCGCCTGGCCTCGAGGCCGCTGCTGCTGGCGCTCCTGTCG<br>TTGGCTCTTTGCCGAGGGGGTCCTATATTTAATGCTTCTGTGCATTCAGACACACCATC<br>AGTAATTCGGGGAGATCTGATCAAATTGTTCTGTATCATCACTGTCGAGGGAGCAGCA<br>CTGGATCCAGATGACATGGCCTTTGATGTGTCCTGGTTTGCGGTGCACTCTTTTGGCCT<br>GGACAAGGCTCCTGTGCTCCTGTCTTCCCTGGATCGGAAGGGCATCGTGACCACCTC<br>CCGGAGGGACTGGAAGAGCGACCTCAGCCTGGAGCGCGTGAGTGTGCTGGAATTCT<br>TGCTGCAAGTGCATGGCTCCGAGGACCAGGACTTTGGCAACTACTACTGTTCCGTGA<br>CTCCATGGGTGAAGTCACCAACAGGTTCCTGGCAGAAGGAGGCAGAGATCCACTCCA<br>AGCCCGTTTTTATAACTGTGAAGATGGATGTGCTGAACGCCTTCAAGTATCCCTTGCT<br>GATCGGCGTCGGTCTGTCCACGGTCATCGGGCTCCTGTCCTGTCTCATCGGGTACTGC<br>AGCTCCCACTGGTGTTGTAAGAAGGAGGTTCAGGAGACACGGCGCGAGCGCCGCAG<br>GCTCATGTCGATGGAGATGGACGGAGGCGGTGGAAGCGGAGGCGGTGGAAGCGGAG<br>GCGGTGGAAGCGCTTCAAACTTTACTCAGTTCGTGCTCGTGGACAATGGTGGGACAG<br>GGGATGTGACAGTGGCTCCTTCTAATTTCGCTAATGGGGTGGCAGAGTGGATCAGCTC<br>CAACTCACGGAGCCAGGCCTACAAGGTGACATGCAGCGTCAGGCAGTCTAGTGCCCA<br>GAAGAGAAAGTATACCATCAAGGTGGAGGTCCCCAAAGTGGCTACCCAGACAGTGG<br>GCGGAGTCGAACTGCCTGTCGCCGCTTGGAGGTCCTACCTGAACATGGAGCTCACTA<br>TCCCAATTTTCGCTACCAATTCTGACTGTGAACTCATCGTGAAGGCAATGCAGGGGCT<br>CCTCAAAGACGGTAATCCTATCCCTTCCGCCATCGCCGCTAACTCAGGTATCTACTAGG<br>ATATC

Table S2. miRNA sequences used in the study.

miRNA	Sense	Antisense
miR-122 mimics	5'UGGAGUGUGACAAUGGUGUUUG3'	5'AACACCAUUGUCACACUCCAUU3'
NC	5'UUCUCCGAACGUGUCACGUTT3'	5'ACGUGACACGUUCGGAGAATT3'
miR-122 inhibitor	5'CAAACACCAUUGUCACACUCCA3'	
inhibitor NC	5'CAGUACUUUUGUGUAGUACAA3'	

Table S3. Primers used in the study:

Primer	Forward	Reverse
mouse *Bax*	5'TGCTACAGGGTTTCATCCA3'	5'AAGTAGAAGAGGGCAACCAC3'
mouse $p16^{inK4a}$	5'CGCAGGTTCTTGGTCACTGT3'	5'TGTTCACGAAAGCCAGAGCG3'
mouse *p21*	5'CCTGGTGATGTCCGACCTG3'	5'CCATGAGCGCATCGCAATC3'
mouse *Tnfα*	5'CTGAACTTCGGGGTGATCGG3'	5'GGCTTGTCACTCGAATTTTGAGA3'
mouse *Ilα*	5'TCCATAACCCATGATCTGGAA3'	5'TTGGTTGAGGGAATCATTCAT3'
mouse *Mmp3*	5'CAAAACATATTTCTTTGTAGAGGACAA3'	5'TTCAGCTATTTGCTTGGGAAA3'
mouse *Mmp13*	5'-AAGGGGATAACAGCCACTACAA-3'	5'-ACCAACATAAAAATTAAGCCAAATG-3'
mouse *Gapdh*	5'AGGTCGGTGTGAACGGATTTG3'	5'GGGGTCGTTGATGGCAACA3'
Human GAPDH	5'CAATGACCCCTTCATTGACC3'	5'GACAAGCTTCCCGTTCTCAG3'
miR-122-5p	5'TGGAGTGTGACAATGGTGTTTG3'	Provided in the Kit
miR-122 inhibitor	5'CAAACACCATTGTCACACTCCA3'	Provided in the Kit
U6	5'CTCGCTTCGGCAGCACA3'	5'AACGCTTCACGAATTTGCGT3'

Supplementary Figures and Figure Legends:

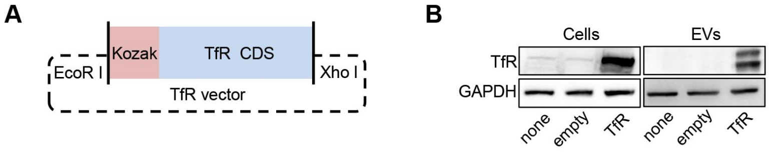

Figure S1. Engineering of TfR modified EV (EV^{TfR}). (A) Schematic diagram of TfR vector. (B) Western blot analysis was performed to detect the expression of TfR protein in parental cells and derived EVs. The cells were transfected with either none, empty vector, or TfR vector, and GAPDH was used as the loading control.

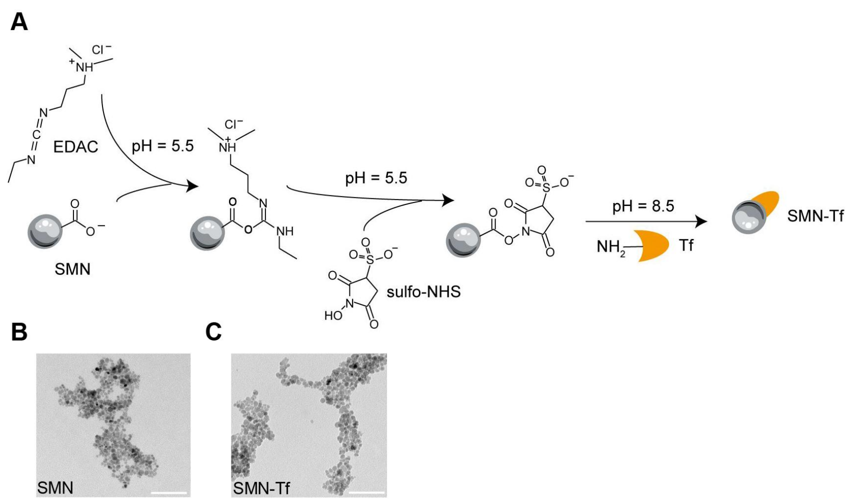

Figure S2. Schematic illustration of the SMN-Tf conjugation procedure and its characterization. (A) The COO^{-} groups located on the surface of SMN were activated using EDAC and sulfo-NHS to facilitate their interaction with the NH^{3+} of Tf. This process led to the successful attachment of Tf to the surface of SMN, ultimately producing SMN-Tf. (B) Representative TEM images of SMN. Scale bar = 100 nm. (C) Representative TEM images of SMN-Tf. Scale bar = 100 nm.

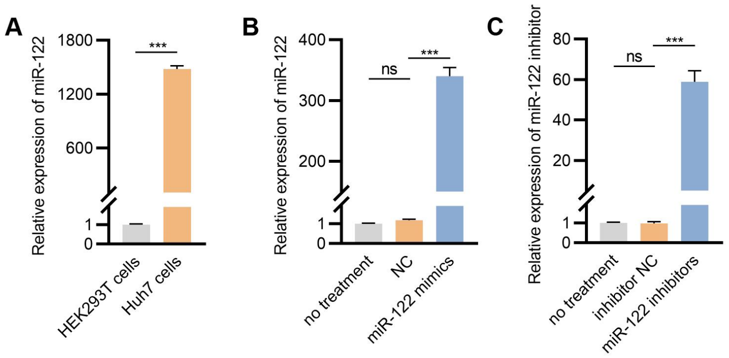

Figure S3. Expression of miR-122 in indicated cells. (A) miR-122 expression levels were compared between HEK293T and Huh7 cells using qPCR analysis, with U6 serving as an internal control. (B) The level of miR-122 expression was analyzed by qPCR in HEK293T cells transfected as indicated, with U6 as an internal control. (C) The level of miR-122 inhibitor expression was analyzed by qPCR in Huh7 cells transfected as indicated, with U6 serving as an internal control. Data are presented as mean ± SEM of three independent experiments. ***$p < 0.001$ by the student's *t*-test or one-way ANOVA with Tukey's post hoc test. ns, no significance.

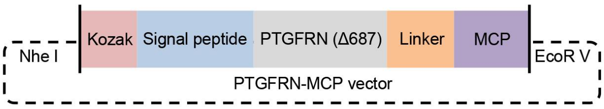

Figure S4. Schematic diagram of PTGFRN-MCP plasmid.

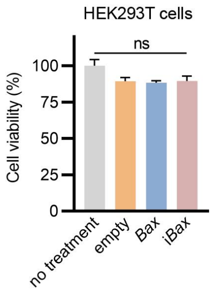

Figure S5. Cell viability in cells with indicated treatments. The cell viability of HEK293T cells was measured by CCK-8 assay after different treatments as indicated. Data are represented by mean ± SEM of three independent experiments. Statistical significance was determined by one-way ANOVA with Tukey’s post hoc test. ns, no significance.

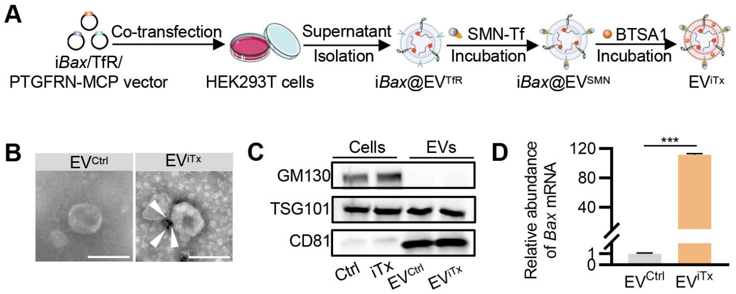

Figure S6. Construction and characterization of EV^{iTx}. (A) Schematic illustration of EV^{iTx} synthesis. HEK293T cells were co-transfected with the PTGFRN-MCP vector, i*Bax* vector, and TfR vector to obtain engineered i*Bax*@EV^{TfR}, which were then incubated with SMN-Tf to create i*Bax*@EV^{SMN}. BTSA1 was then membrane-loaded onto i*Bax*@EV^{SMN}, resulting in EV^{iTx}. (B) Representative TEM images of EV^{iTx}. Scale bar = 100 nm. White arrows are SMN-Tf. (C) Western blot analysis was performed to examine exclusive and inclusive markers in both isolated EVs and parental cells. Ctrl: HEK293T cells transfected with empty vector; iTx: HEK293T cells were co-transfected with the i*Bax* vector, PTGFRN-MCP vector, and TfR vector. (D) qPCR analysis of the loading efficacy of *Bax* mRNA of EVs as indicated. Data are presented as mean ± SEM of three independent experiments. ***p < 0.001 as determined by student's t-test.

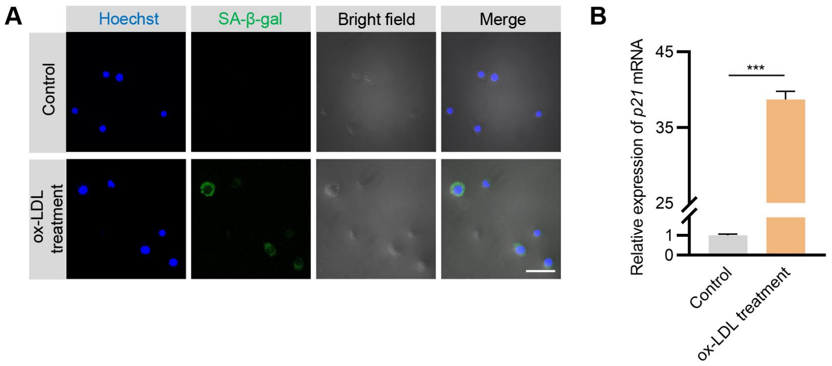

Figure S7. ox-LDL treatment induces senescent phenotype in macrophages. (A) *In vitro* SA-β-gal staining of macrophages stimulated with ox-LDL. Scale bar = 50 μm; (B) qPCR analysis of *p21* mRNA expression in macrophages that were either stimulated with ox-LDL or left unstimulated. GAPDH served as an internal control. Data are presented as mean ± SEM of three independent experiments. $***p < 0.001$ as determined by the student's *t*-test.

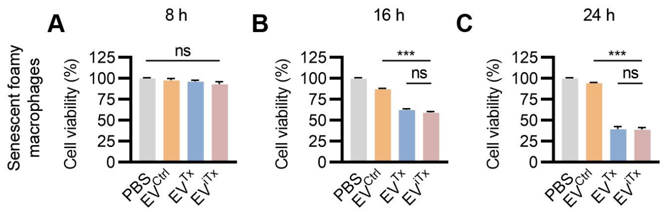

Figure S8. Cell viability in senescent foamy macrophages with indicated treatments. The cell viability of senescent foamy macrophages following the indicated treatments was assessed at multiple time points: (A) 8 h, (B) 16 h, and (C) 24 h. Data are represented by mean ± SEM of three independent experiments. Statistical significance was determined by one-way ANOVA with Tukey's post hoc test. ***p < 0.001, ns, no significance.

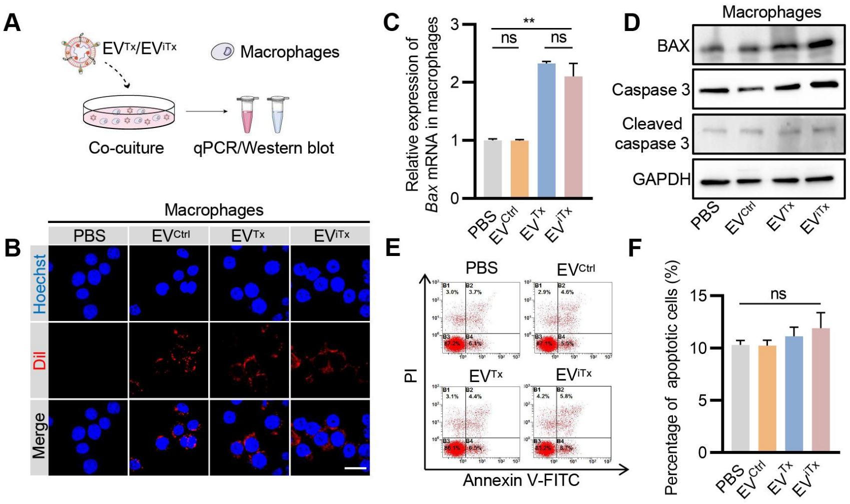

Figure S9. EV^{iTx} induces no obvious apoptosis in healthy macrophages. (A) Schematic of EVs co-cultured with macrophages. (B) Uptake of EVs in macrophages. Scale bar = 20 μm. (C) qPCR analysis of *Bax* mRNA in macrophages as indicated. GAPDH served as an internal control. (D) Western blot analysis was performed to evaluate BAX or cleaved caspase-3 protein expression in macrophages treated with various EVs, as indicated. GAPDH was used as the loading control. (E) Flow cytometry detection of apoptotic macrophages. (F) Quantitative analysis of Figure S9E. Data are represented by mean ± SEM of three independent experiments. Statistical significance was determined by one-way ANOVA with Tukey's post hoc test. $^{**}p < 0.01$, ns, no significance.

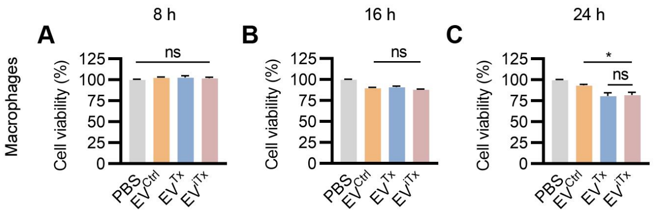

Figure S10. Cell viability in healthy macrophages with indicated treatments. The cell viability of healthy macrophages following the indicated treatments was assessed at multiple time points: (A) 8 h, (B) 16 h, and (C) 24 h. Data are represented by mean ± SEM of three independent experiments. Statistical significance was determined by one-way ANOVA with Tukey's post hoc test. *$p < 0.05$, ns, no significance.

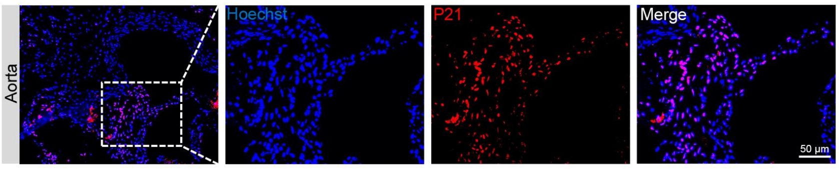

Figure S11. Abundant senescent cells in the aortic root in high-fat diet ApoE$^{-/-}$ mice. Immunofluorescence staining of the P21 expression (in red) in aortic roots from high-fat diet ApoE$^{-/-}$ mice. Nuclei were stained with Hoechst (in blue).

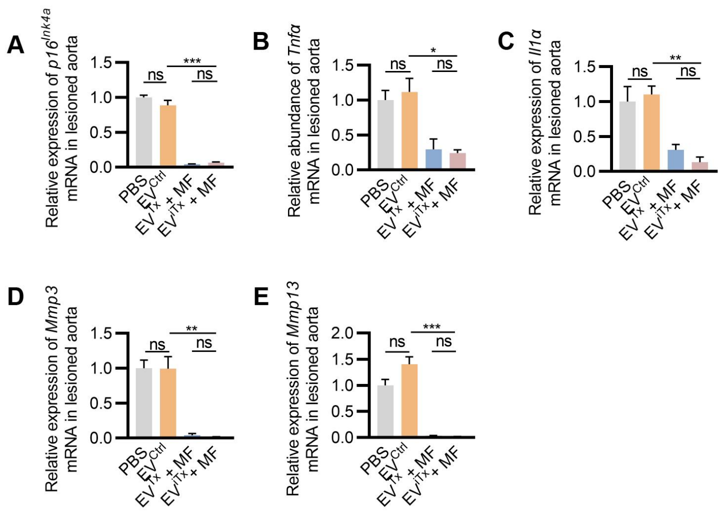

Figure S12. Expression of *p16^{Ink4a}* and SASP factors in the lesioned aorta. (A-E) qPCR analysis of *p16^{Ink4a}* mRNA (A), *Tnfα* mRNA (B), *Il1α* mRNA (C), *Mmp3* mRNA, (D) *Mmp13* mRNA (E) levels in lesioned aorta of high-fat diet ApoE$^{-/-}$ mice receiving indicated treatments. Data are represented by mean ± SEM. n = 6 per group. Statistical significance was determined by one-way ANOVA with Tukey's post hoc test. *$p < 0.05$, **$p < 0.01$, ***$p < 0.001$, ns, no significance.

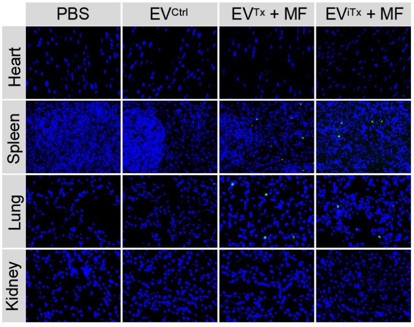

Figure S13. TUNEL staining of the indicated organs from ApoE$^{-/-}$ mice receiving different EVs. Representative images of the TUNEL staining of different organs (heart, spleen, lung, kidney) from the ApoE$^{-/-}$ mice with indicated treatments.

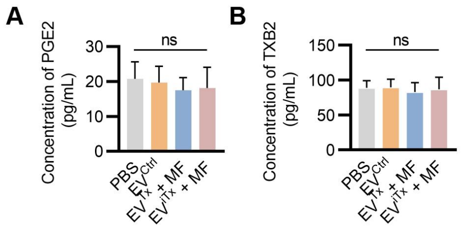

Figure S14. PGE2 (A) and TXB2 (B) level changes in the plasma in ApoE$^{-/-}$ mice receiving different EVs treatments. Data are represented by mean ± SEM. n = 6 per group. Statistical significance was determined by one-way ANOVA with Tukey's post hoc test. ns, no significance.

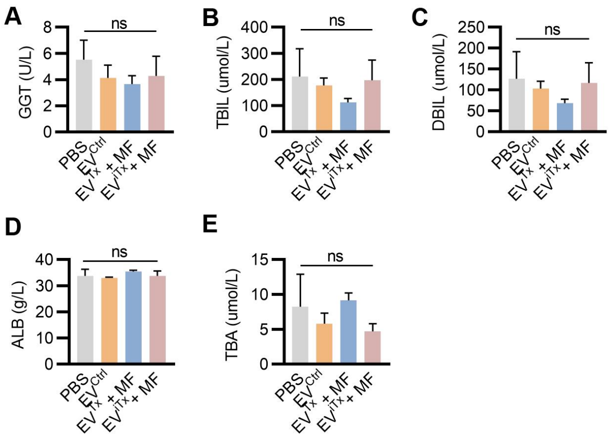

Figure S15. Hepatic function changes observed in ApoE$^{-/-}$ mice receiving different treatments. (A-E) Examination of the GGT (A), TBIL (B), DBIL (C), ALB (D) TBA (E) levels in ApoE$^{-/-}$ mice was conducted among the indicated groups. GGT, γ-glutamyl transpeptadase; TBIL, Total bilirubin; DBIL, Direct bilirubin; ALB, Albumin; TBA, Total bile acid. Data are represented by mean ± SEM. n = 6 per group. Statistical significance was determined by one-way ANOVA with Tukey's post hoc test. ns, no significance.

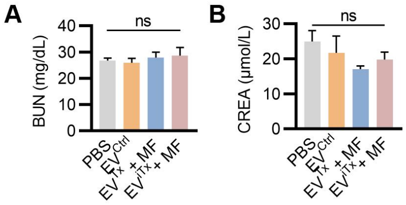

Figure S16. Kidney function changes observed in ApoE$^{-/-}$ mice receiving different EVs treatments. (A-B) Examination of the BUN (A), and CREA (B) levels in ApoE$^{-/-}$ mice was conducted among the indicated groups. BUN, Blood urea nitrogen; CREA, Creatinine. Data are represented by mean ± SEM. n = 6 per group. Statistical significance was determined by one-way ANOVA with Tukey's post hoc test. ns, no significance.

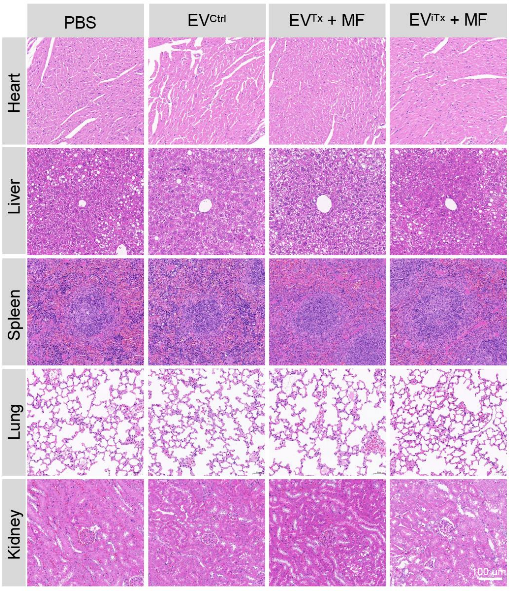

Figure S17. Histological changes of the indicated organs from ApoE$^{-/-}$ mice receiving different EVs. H&E staining of vital organs (heart, liver, spleen, lung, kidney).

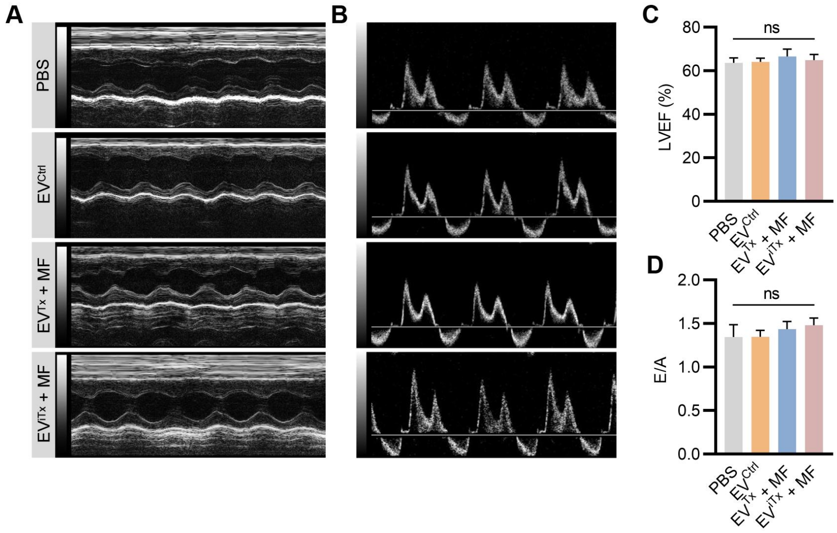

Figure S18. Cardiac function changes in ApoE$^{-/-}$ mice receiving different EVs treatments. (A) Representative images of M mode echocardiography of ApoE$^{-/-}$ mice in the indicated groups. (B) Representative images of mitral flow Doppler echocardiography. (C) Quantification of systolic function parameter LVEF. (D) Quantification of diastolic function parameter E/A. Data are represented by mean ± SEM. n = 6 per group. Statistical significance was determined by one-way ANOVA with Tukey's post hoc test. ns, no significance.
